# Long-reads metagenomics reveals the effects of dulse supplementation on the poultry caecal bacteriome and its associated genetic repertoire

**DOI:** 10.3389/fmicb.2026.1868730

**Published:** 2026-07-15

**Authors:** Julio Cesar Ortega Cambara, Piotr Cuber, Farina Khattak, Pedro Humberto Lebre, Salvatore Galgano, Jos Houdijk, Duncan Smallman, Patricia Estridge, Michael J. Allen, Fiona Short, Martin Sutcliffe, Hermine V. Mkrtchyan

**Affiliations:** 1 Centre for Innovation in Genomics and Microbiome Sciences (CIGMiS), School of Medicine and Biological Sciences, University of West London, London, United Kingdom; 2 School of Veterinary Medicine and Biosciences, Monogastric Science Research Centre, Scotland’s Rural College, Edinburgh, United Kingdom; 3 Centre for Microbial Ecology and Genomics, University of Pretoria, Pretoria, South Africa; 4 Seaweed Generation Ltd, Cornwall, United Kingdom; 5 Department of Biosciences, Faculty of Health and Life Sciences, University of Exeter, Exeter, United Kingdom; 6 UK Agri-Tech Centre, Innovation Centre, York, United Kingdom

**Keywords:** antibiotic resistance genes, chicken caecal bacteriome, dulse dietary interventions, mobile genetic elements, virulence factors

## Abstract

**Introduction:**

Dulse (*Palmaria palmata*) is a macroalgal feed ingredient rich in polysaccharides and bioactive compounds that offers a sustainable strategy to enhance animal health and productivity through modulation of gut microbiota. However, the impact of dulse supplementation on the taxonomic composition and genetic repertoire of the broiler chicken caecal microbiota remains poorly characterised.

**Methods:**

We applied long-read shotgun metagenomic sequencing on 18 caecal samples collected from 27-day-old male Ross 308 broilers following a 7-day feeding trial with three dietary treatments — a reference diet, a soyabean meal-supplemented diet, and a diet supplemented with 30% dulse — to investigate the effects of dulse inclusion on microbial community composition, genetic diversity, and antimicrobial resistance (AMR) and virulence determinants.

**Results:**

Across all dietary treatments, the *Clostridia* class predominated (71%), whereas primary fermenters (*L. phocaeense*), lactic acid bacteria (*L. salivarius*), and hydrogenotrophic cross-feeders (*B. hydrogenotrophica*) were enriched in the reference diet, dulse-supplemented and soyabean meal-supplemented groups, respectively (KW *p* < 0.05), contributing to potential improvements in caecal function, immune resilience, and nutrient utilisation while reducing pathogen load. The overall resistome profiles were comparable across dietary treatments and were dominated by genes conferring resistance to tetracyclines, lincosamides, and aminoglycosides. In contrast, the virulome displayed diet-associated shifts: *Enterobacteriaceae* were enriched in the dulse and reference diets relative to the soyabean meal diet, with an expanded functional repertoire of virulence-associated genes, particularly those involved in adhesion, iron acquisition, and secretion systems. Multidrug resistance genes, virulence determinants, and *Col*/*IncF*-type plasmid replicons were associated with *E. coli* reads, highlighting its potential resistance and virulence arsenal within the caecal microbiota.

**Discussion:**

Our findings suggest that the benefits of dulse extend beyond its nutritional value, residing in its ability to foster ecosystem resilience; by promoting a diverse, niche-stabilised microbiota, dulse minimises the risk of opportunistic pathogen proliferation, supporting its use as a sustainable, functional feed ingredient.

## Introduction

The gastrointestinal tract (GIT) of poultry harbors a complex and metabolically diverse microbial ecosystem that plays a pivotal role in nutrient absorption, immune modulation, and protection against enteric pathogens (Hussain et al., 2024). Both the taxonomic composition and functional activity of this microbiota are profoundly influenced by diet, which provides substrates for microbial metabolism and shapes intestinal homeostasis (Pan and Yu, 2014). Consequently, dietary interventions have become a key strategy to optimise gut health and performance while reducing dependence on in-feed antimicrobials in intensive poultry production systems (Oni and Oke, 2025; Adedokun and Olojede, 2019; Diarra and Malouin, 2014). However, the GIT also constitutes a major reservoir for a wide array of antimicrobial resistance (AMR) genes and virulent determinants, which can be horizontally transferred among microbial populations (Yang et al., 2022). Understanding the co-occurrence and mobility of these genetic elements is therefore essential for assessing their potential contribution to zoonotic transmission. Recent advances in high-throughput sequencing have greatly expanded our knowledge of the poultry gut microbiota, revealing its critical role in maintaining host health, enhancing productivity, and modulating disease susceptibility (Kers et al., 2018). In addition to AMR, the colonisation of poultry gut by zoonotic pathogens such as *Salmonella*, *Clostridioides*, *Klebsiella*, *Campylobacter*, and pathogenic *Escherichia* continues to pose significant public health and food safety challenges (Sharma S. et al., 2025; Sharma I. et al., 2025; Singh et al., 2025). These pathogens frequently persist asymptomatically in the avian intestinal tract; consequently, modulating the gut microbiota through nutritional strategies offers a promising approach to limit their carriage and transmission (Hejna et al., 2024). To comprehensively evaluate these gut dynamics, this study distinguishes between the bacterial community itself (microbiota) and its collective genetic and functional landscape (microbiome), specifically characterised here via the resistome, virulome, and mobilome (Marchesi and Ravel, 2015; Berg et al., 2020). Given that bacteria constitute the primary drivers of these processes, we focus specifically on the bacteriome fraction utilising high-resolution, long-read shotgun sequencing.

Marine macroalgae (seaweeds) represent sustainable bioresources owing to their high nutritional value, prebiotic polysaccharides, bioactive peptides, and antioxidant compounds (Ganesan et al., 2019; Shannon et al., 2021). Seaweed-derived polysaccharides, including laminarin, carrageenan, and xylans can modulate gut microbiota composition and fermentation activity, promoting the proliferation of beneficial bacteria and short-chain fatty acid (SCFA) production (González-Ortiz et al., 2019; de Jesus Raposo et al., 2016). In addition, seaweed polyphenols and pigments, including phycobiliproteins, have demonstrated antioxidant and mild antimicrobial properties that contribute to mucosal defense and immune regulation (Gupta and Abu-Ghannam, 2011; Moroney et al., 2013; Jayapala et al., 2025; Lee et al., 2025). Understanding these interactions within the GIT of poultry is essential for assessing the potential of seaweed as a functional feed ingredient that supports poultry productivity while contributing to food safety and reducing the risk of zoonotic disease transmission within a One Health framework (Evans and Critchley, 2014; Kulshreshtha et al., 2014).

*Palmaria palmata* (dulse) is a red macroalga rich in protein, xylan-based structural polysaccharides and floridean starch, alongside polyphenols and pigments (Bjarnadóttir et al., 2018; Usov, 2011; Harnedy and FitzGerald, 2013). *β*-(1 → 3)/(1 → 4)-D-xylans are considered as fermentable dietary fibre that can stimulate short-chain fatty acid (SCFA) production while promoting the abundance of beneficial taxa in the caeca, these effects are potentially enhanced by xylo-oligosaccharides (XOS) generated via endogenous or supplemental xylanases (de Jesus Raposo et al., 2016; González-Ortiz et al., 2019). Dulse is also distinguished by its balanced amino acid composition and high mineral content (Kulshreshtha et al., 2014; Lee et al., 2017). Its inclusion in animal feed has been linked to improved growth performance, antioxidant capacity, and gut morphology (Oretomiloye and Adewole, 2024; Liu et al., 2020). However, emerging resistome-focused studies demonstrated that the poultry gut harbors a diverse and dynamic reservoir of antibiotic resistance genes, shaped by diet, antimicrobial exposure, and mobile genetic elements (Koorakula et al., 2022; Yang et al., 2022; Ma et al., 2021). These studies highlight the importance of genomic-scale approaches for understanding how dietary interventions influence microbial function and AMR dissemination. Understanding these interactions is crucial, as the gut microbiota acts as both a driver of nutrient utilisation and a reservoir for resistance and virulence determinants with implications for the wider One Health continuum (Dongre et al., 2025; Velazquez-Meza et al., 2022; Sharma S. et al., 2025; Sharma I. et al., 2025; Maciel-Guerra et al., 2023). Importantly, dulse’s potential to limit pathogen carriage may operate not through pathogen presence or removal alone, but via two coupled mechanisms: restructuring of the resident microbial community to constrain pathogen niches via competitive exclusion and altered fermentation milieu, and modulation of the abundance and mobility of virulence and resistance determinants that underpin pathogen persistence and dissemination.

Despite this nutritional and bioactive profile, evidence on how dulse reshapes the poultry gut microbiome remains limited. What is known is largely confined to performance, antioxidant capacity, and gut morphology outcomes. For instance, dulse inclusion has been associated with improved growth, antioxidant status, and intestinal architecture in broilers (Kulshreshtha et al., 2014; Liu et al., 2020; Oretomiloye and Adewole, 2024). What remains unknown is how dulse modulates the broiler caecal microbiota—specifically, whether it restructures bacterial community composition at the strain level, and whether it alters the abundance, host distribution, and mobility of antimicrobial resistance genes (ARGs), virulence factors (VFs), and mobile genetic elements (MGEs). Its characterisation is consequential because the poultry gut is a recognised reservoir for the dissemination of ARG and VF via mobile elements (Koorakula et al., 2022; Ma et al., 2021; Yang et al., 2022), and dietary interventions designed to modulate its structure may silently drive shifts in these functional landscapes.

To address this knowledge gap, we applied a long-read shotgun metagenomics to broiler caecal samples, collected upon apparent metabolisable energy (AME) trials to investigate the effects of dietary dulse supplementation on the broiler GIT microbiome, comprising both the taxonomic composition of the bacteriome and its associated functional gene repertoire, with a particular emphasis on AMR and virulence determinants (Olukosi et al., 2017, 2019). Long-read sequencing technologies, such as those developed by Oxford Nanopore Technologies (ONT), generate reads that typically exceed 5 kb (often >10 kb) in length. These extended read lengths preserve the physical linkage between taxonomic markers and adjacent functional genetic elements on the same read. As a result, long-read sequencing enables direct attribution of functional genes to their host organisms, overcoming a key limitation of short-read approaches, which often fragment genomes into multiple unlinked contigs and obscure such associations. Furthermore, the enhanced contiguity and strain-level resolution provided by long reads facilitate the detection of fine-scale, diet-induced shifts in both community composition and genomic variation. This is particularly valuable in relatively stable microbial ecosystems, such as caecal communities, where subtle changes at the strain level may not be apparent through conventional sequencing approaches. To our knowledge, this study is the first long-read metagenomic evaluation of caecal microbiota upon inclusion of dulse broiler diets.

## Materials and methods

### Animal experiment and sample collection

Caecal samples were obtained from a parallel *in vivo* metabolism experiment designed to determine the apparent metabolisable energy of dulse in broiler chickens following the approach described by (Olukosi et al., 2017, 2019). Male Ross 308 broilers were reared under controlled environmental conditions in accordance with breed management guidelines and Home Office regulations (Aviagen, 2022). At 20 days of age, birds were transferred to metabolism cages and allocated to three dietary treatments in a completely randomised block design, with six replicates per treatment and five birds per cage.

The experimental diets, formulated for AME determination, consisted of a wheat–soyabean meal reference diet (65.65% wheat and 26.95% soyabean meal), a soyabean-meal–enriched diet produced by replacing 30% of the reference diet with additional soyabean meal, and a dulse diet in which 30% of the reference diet was replaced with dried *Palmaria palmata*. All diets were formulated to be iso-nitrogenous and iso-energetic and were offered ad libitum.

After a seven-day feeding period, birds were humanely euthanised by cervical dislocation, in accordance with UK Home Office Schedule 1 procedures and institutional ethical approval; and caecal digesta were aseptically collected, pooled per cage replicate (*n* = 6 per treatment), and stored for long-read metagenomic sequencing. All animal procedures were conducted under the Animal Scientific Procedures Act (1986) with approval from the SRUC Ethical Review Committee (AEX 2024 -021POU).

### DNA extraction and purification

Genomic DNA was extracted from the 18 chicken caecal samples (approximately 200 mg digesta/sample) using the ZymoBIOMICS™ DNA Miniprep Kit (Zymo Research, Cambridge, UK),following the manufacturer’s protocol. The method combines mechanical and chemical lysis to effectively recover DNA from complex microbial communities (Zymo Research, 2025). DNA purification was performed using Zymo-Spin™ column technology, to obtain inhibitor-free DNA suitable for downstream metagenomic sequencing and functional analysis.

### Shot-gun whole metagenomics sequencing

The extracted gDNA (200 ng per sample) was sequenced for 72 h using ONT’s Rapid Barcoding (SQK-RBK114.24) protocol, real-time MinION platform (Oxford Nanopore Technologies, Oxford, UK). Samples were multiplexed to reduce the cost of sequencing. The libraries were loaded on the R10.4.1 flow cells using MinION (FLO-MIN106D) device. Live basecalling was carried out using Dorado v0.7.3 in super-accurate mode. The epi2me-labs/workflow v2.10.1 was run offline to validate the metagenomics sequencing.

### Bioinformatic analysis

We used an in-house metagenomic pipeline that includes several key steps, from sample processing to data analysis, such as taxonomic classification, relative abundance assessment, contaminants removal, and the linkage of microbial community to antibiotic resistance genes (ARGs), virulence factors (VFs), and mobile genetic elements (MGEs) profiles.

Prefiltering, taxonomic annotation and relative abundance: First, only the raw reads that have passed internal data quality thresholds (Q-score > 10) during sequencing (“passed” sequencing reads) were processed. For reproducibility, a quality control check was performed using FastQC v0.12.1, adapter removal with porechop v0.2.4, filtering of low complexity and quality reads with Filtlong v0.2.1, and host-read removal through Minimap2 v2.24 (Li, 2018) by mapping the reads against the chicken reference genome (*Gallus gallus* Annotation Release 106, accession: GCF_016699485.2) from National Center for Biotechnology Information (NCBI) database. The pipeline also supports statistics for host-read removal (using Samtools v1.17) and conducts taxonomic classification and/or profiling with Kraken2 v2.1.2 (Wood et al., 2019) using the PlusPF-8 database (accessed Apr 26, 2025), which includes RefSeq sequences for archaea, bacteria, protozoa, fungi, and viruses, alongside the human genome and UniVec_Core sequences (Ewels et al., 2020). The relative abundance was estimated with Bracken v2.7.0 (Supplementary file 1), and the taxa exploration was carried out with Krona v2.8.1 tools.

ARGs, VFs and MGEs detection: After taxonomy classification with Kraken2, the classified reads were screened with ABRicate v1.0.1, employing the CARD (Jia et al., 2017), VFDB (Chen et al., 2016), and PlasmidFinder (Carattoli et al., 2014) databases to identify antibiotic resistance genes (ARGs), virulence factors (VFs), and mobile genetic elements (MGEs), respectively. Contaminants were subsequently removed using re-centrifuge (Martí, 2019), which implements a robust method for the removal of negative controls and crossover taxa from the rest of the samples. The reads count from potential contaminants was retrieved from the metagenomics dataset. Subsequently, we combined the Kraken2 report with the ABRicate summary report using a customised R script, establishing a direct read-level association between each detected genetic element and its host taxon: each ARG, VF, or MGE was identified by ABRicate on the same long read that Kraken2 had taxonomically classified. Taxon-GE pairs, therefore, reflect physical co-residence on the same sequencing read, rather than sample-level co-occurrence alone.

Genetic elements (GEs) count normalisation: Raw read counts attributed to each GE were normalised as transcripts per million (TPM; Supplementary file 2) using a custom R script. For each GE in samples, reads per kilobase (RPK) were first computed by dividing the raw read count by the GE length in kilobases, where GE length was derived from the ABRicate alignment coordinates as (END − START + 1). A per-sample scaling factor was then applied so that TPM values within each sample sum to 10^6^, computed as: TPM = RPK/(*Σ* RPK/10^6^), where Σ RPK denotes the sum of RPK values across all GEs in the same sample. TPM was selected because it simultaneously corrects for sequencing depth and gene length and, unlike RPKM, sums to a constant per sample, enabling direct between-sample comparison of relative GE abundance (Wagner et al., 2012; Pereira-Marques et al., 2019). While TPM was originally developed for transcriptomics, its application to metagenomic gene quantification is increasingly common (Koorakula et al., 2022). For downstream visualisation, TPM values were additionally rescaled to per-sample relative abundances by dividing by the per-sample TPM total.

Alpha and Beta Diversity analysis: We measured alpha diversity metrics within each treatment in terms of taxa and genetic elements richness (i.e., number of unique taxa/elements represented) and diversity (Shannon index, i.e., abundance and prevalence among each taxa/element). Beta diversity was further assessed by using the Bray–Curtis dissimilarity index, and differences in taxonomic and genetic element composition among treatment groups were evaluated via PERMANOVA (adonis2, 9,999 permutations, *p* < 0.05). Ordination was conducted using Principal Coordinates Analysis (PCoA) to visualise multivariate patterns and treatment-associated clustering.

Differential abundance (DA) analysis: DA analysis was implemented through a custom R script designed to automate ALDEx2-based differential abundance testing (Fernandes et al., 2014). The pipeline imported species-level count tables and sample metadata, aligned sample identifiers, and filtered low-prevalence taxa (no. of samples < 2) before performing DA comparisons across dietary treatments using ALDEx2 with 128 Monte-Carlo Dirichlet instances (Fernandes et al., 2014). For each comparison, the effect sizes, overlap metrics, and empirical Bayes Benjamini-Hochberg (eBH) corrected *p*-values were generated. Complementary, per-taxon mean and standard error of raw counts within each treatment group were also estimated. Finally, the workflow identified high-effect taxa across pairwise treatment comparisons using a defined threshold (effect > 0.8, overlap < 0.2) and generated a DA summary table (Supplementary file 3).

Microbial network analysis: The network was constructed using a bipartite graph with several nodes derived from the study data (N samples, T taxa, G genetic elements). The connections (edges) were defined as follows: Sample–Taxa Edges: Undirected edges linked individual samples to specific bacterial taxa present within that sample. Edge weights were proportional to the relative abundance of the corresponding taxon. Taxa–GEs Edges: Undirected-unweighted (binary presence/absence) edges linked bacterial taxa to the associated ARGs, VFs, or MGEs. The network was imported into Gephi software (v. 0.10) for spatialisation and visualisation (Bastian et al., 2009). The Fruchterman-Reingold force-directed layout algorithm was applied to arrange the nodes in a 2D space (Fruchterman and Reingold, 1991). The algorithm was iterated until the network reached a minimal energy state, facilitating the visual identification of clusters associated with a specific treatment. Node colours and size were assigned based on their type [e.g., taxa (functional groups), GEs (ARG, VF, MGE)] and experimental group affiliation. Network topology metrics (degree centrality, betweenness, and modularity) were also calculated to identify central hubs in the caecal microbial ecosystem.

### Statistical analysis

All statistical analyses were conducted using the vegan package (version 2.6.10) in R environment (4.5.0). Statistical significance was defined as (*p* < 0.05) using the non-parametric Kruskal–Wallis test to determine if there is an overall difference among the three diet groups, and the Wilcoxon rank-sum test (Mann–Whitney *U* test) was used for post-hoc pairwise comparisons between each specific pair of groups. *p*-values were adjusted for multiple comparisons using the Benjamini-Hochberg (BH) method, and all results are reported with their corresponding *p*-values. The Venn diagrams were created using the “VennDiagram” package (1.7.3), heatmaps were generated with the “pheatmap” package (1.0.12), and the chord diagram was produced with the “circlize” package (0.4.16) (Gu et al., 2014). The remaining figures were created using the “ggplot2” package (3.5.2) and coloured with the “paletteer” package (1.6.0). Exploration of the chicken caecal microbiota and the genetic makeup was conducted using the R shiny (1.12.1) application called “chickMicro,” developed as part of this research project.^1^

## Results

### Taxonomic profile and relative abundance analysis of poultry caecal bacteriome

#### Taxonomy profile

**Figure 1:**
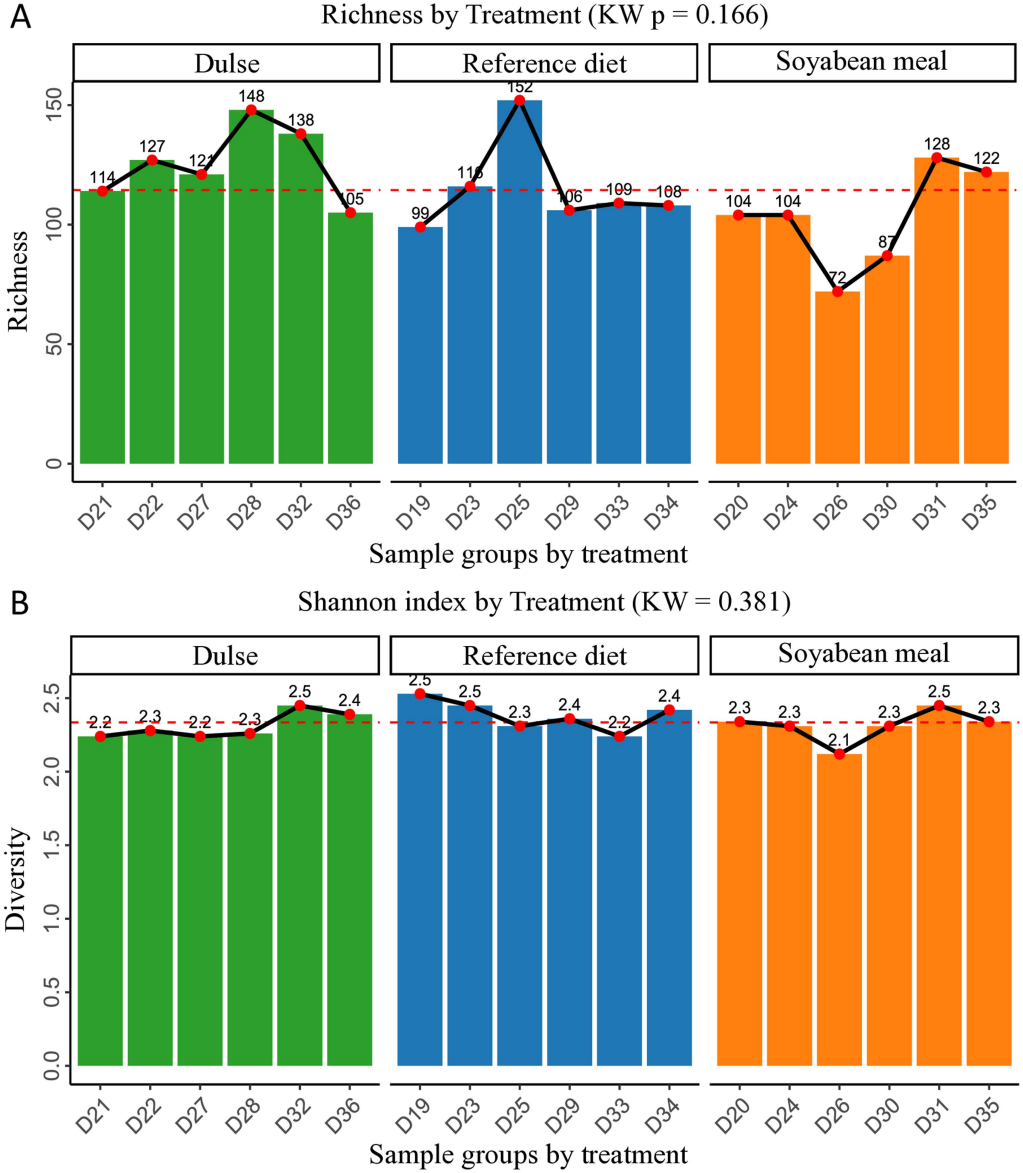
Alpha diversity metrics measured as Richness **(A)** and **(B)** diversity (Shannon index) of taxa across treatments and over sampling points. Bars and red dots represent the index value per sample. The horizontal red line represents the median. Significance was tested with a Kruskal–Wallis test (*p* < 0.05). Colours indicated different treatments.

**Figure 2:**
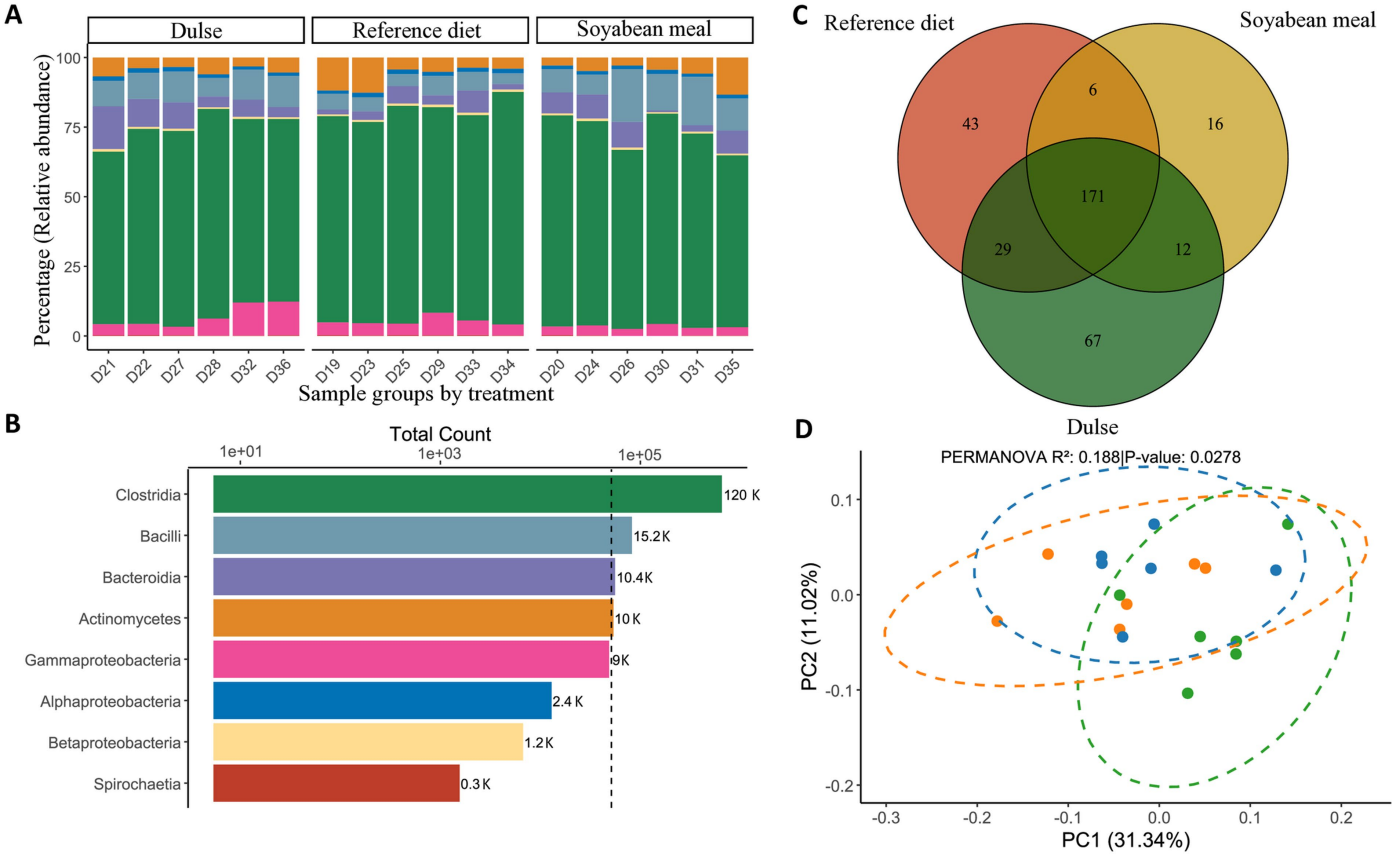
Taxonomy diversity and relative abundance profile of the poultry caecal bacteriome across three dietary treatments. **(A)** Relative abundance of major bacterial classes across individual samples grouped by treatment. Each bar represents a sample, with colours indicating taxonomic classes. **(B)** Total count of bacterial classes aggregated across all samples. **(C)** Venn diagram showing shared and unique taxa among treatments. **(D)** Principal Coordinates Analysis (PCoA) of samples based on Bray–Curtis dissimilarities of the bacteriome profile, and differences in species-level composition among treatments were assessed with PERMANOVA (adonis2, 9,999 permutations).

With the read counts per taxon, we assessed the relative abundance, richness, and diversity of taxa across all treatment groups (Supplementary file 1). The analysis of alpha diversity metrics showed that the total number of identified genera (richness) and the evenness of their abundance (Shannon diversity) were not statistically different (KW *p* > 0.05) across dietary treatments (Figures 1A,B). While pairwise comparisons did not identify significant differences between any treatment pairs, a higher mean richness was found in the Dulse group (126 ± 6.42), followed by the Reference diet (115 ± 7.73) and slightly lower in the Soyabean meal (103 ± 8.57) group. Microbial community profiling revealed a conserved taxonomic profile across all dietary treatments, with *Clostridia* class consistently dominating the caecal bacteriome, accounting for approximately 71% (*n* = 120,829/169366) of the total long-read contigs classified at the class level (Figures 2A,B); followed by *Bacilli* (8%, *n* = 15,227), *Bacteroidia* (6.1%, *n* = 10,352), *Actinomycetes* (5.9%, 9,986) and *Gammaproteobacteria* (5.3%, *n* = 9,029). By comparing taxa that are found across all groups at the species level, a total of 171 shared taxa were identified, representing a common microbiome among diets (Figure 2C). Beta-diversity across treatment groups based on Bray–Curtis dissimilarity distance matrix revealed a clear overlap among all treatment-specific clustering (PERMANOVA *R*^2^ = 0.188, *p* = 0.0278), indicating that diet does have a limited, yet significant effect on the species-level composition of the caecal bacteriome (Figure 2D). Notably, the Dulse supplemented group (diet 3) exhibited the highest number of unique taxa (*n* = 67), followed by the Reference diet (*n* = 43) and Soyabean meal (*n* = 16) groups. However, most taxa identified as unique to each dietary treatment appeared to be infrequent or transient members of the microbial community, suggesting they were rare rather than being modulated by specific dietary factors (Supplementary Figure S1). This inference was supported by two observations from the relative-abundance profile: (1) many unique taxa were detected in a single sample and had low relative abundance (<0.5% of read counts), and (2) the unique taxa were related to transient members (environmental and/or opportunistic lineages) that commonly occur at low-abundance.

Moreover, the taxa shared between Dulse and Reference diet groups included members of the phyla *Firmicutes* and *Bacteroidetes*, including key short-chain fatty acid (SCFA)-producing genera such as *Ruminococcus* species (*R. cellulolyticum*, *R. bovis*) and *Clostridium* species (*C. thermocellus*), along with lactic acid bacteria/probiotics (LAB/P) such as *Lactobacillus* species (*L. crispatus*, *L. reuteri*, etc.). Overall, the Dulse and Reference diet samples exhibited a greater degree of similarity in their overall microbial composition than the Soyabean meal diet samples (Supplementary Table S1, Figure S2).

#### Relative abundance

**Figure 3:**
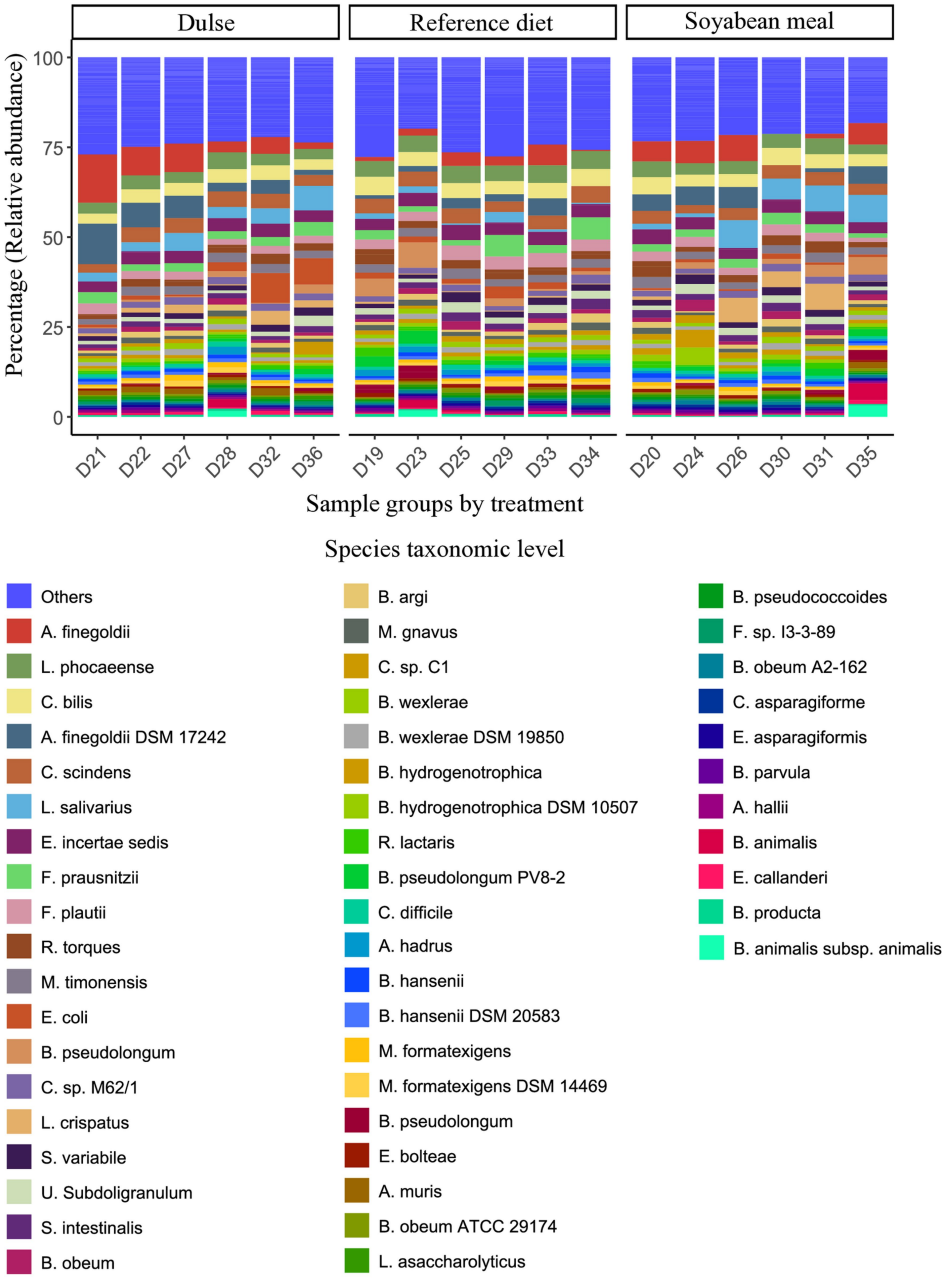
The figure illustrates the top 50 most abundant taxa across all chicken caecal samples; the *y*-axis represents the relative abundance at the species level in descending order, and the *x*-axis represents the sample groups by treatment.

The top 50 taxa collectively accounted for 75% of the relative abundance across all sample groups, revealing a microbial community that was dominated by obligatory anaerobic fermenters, SCFA producers and LAB/P (Figure 3; Supplementary Table S1), indicating a conserved metabolic backbone in the caecal bacteriome. Among them, genera present in all treatments included *Alistipes*, *Faecalibacterium*, *Blautia*, *Ruminococcus*, *Subdoligranulum*, *Anaerostipes*, *and Anaerobutyricum* (SCFA producers), as well as members of the *Bifidobacterium*, *Lactobacillus*, and *Ligilactobacillus* groups (LAB/P). Furthermore, differential abundance analysis (DAA) among treatments was carried out using ALDEx2 (Supplementary file 3). A total of 30 taxa at the species level were identified with moderate to large, centred log ratio (CLR) effect sizes (effect > 0.8) and low distributional overlap (< 0.2) (Table 1). However, after multiple test corrections, no taxa reached a false discovery rate (FDR) < 0.05, possibly due to the discrete sample size and short collection period of the study design. Relative abundance of these 30 taxa further highlighted that only 12 taxa were prevalent across all sample groups [0.4–0.1], and the rest had low abundance [0–0.2] (Supplementary Figure S3).

**Table 1:** Summary table of the top candidates selected from the differential abundance analysis (DAA).

Taxon	Overall ALDEx2: kw p^a^/kw eBH^b^	Effect^c^/overlap^d^			Mean ± SE		
		Dulse vs reference	Reference vs soyabean	Dulse vs soyabean	Dulse	Reference diet	Soyabean meal
*L. phocaeense*	0.036/0.52	1.42/0.10	−0.83/0.20	0.47/0.29	368.83 ± 67.97	397.5 ± 65.5	266 ± 54.18
*L. salivarius*	0.035/0.53	−1.20/0.08	1.04/0.16	0.32/0.36	408.17 ± 63.04	103.5 ± 32.8	408.33 ± 152.59
*F. plautii*	0.009/0.49	1.88/0.02	−0.89/0.13	0.42/0.31	226.33 ± 23.16	291.7 ± 63.23	162.5 ± 26.49
*E. coli*	0.059/0.56	0.07/0.49	−1.42/0.07	−0.72/0.25	380.17 ± 176.9	146.67 ± 28.22	52.33 ± 9.28
*L. crispatus*	0.087/0.59	−1.36/0.08	0.78/0.28	0.19/0.44	189.33 ± 78.1	22 ± 3.87	222.5 ± 126.28
*U. Subdoligranulum*	0.168/0.65	0.86/0.22	−0.03/0.49	0.49/0.78	130.5 ± 19.39	174.17 ± 50.83	129 ± 26.75
*S. intestinalis*	0.076/0.58	0.88/0.17	0.05/0.48	0.97/0.13	133.67 ± 18.45	167.17 ± 42.01	121.83 ± 17.54
*B. hydrogenotrophica*	0.020/0.50	0.78/0.22	0.57/0.21	1.26/0.03	78.33 ± 7.25	82.67 ± 15.66	127 ± 41.83
*E. bolteae*	0.082/0.57	0.88/0.14	−0.01/0.46	0.76/0.18	65.17 ± 10.33	72 ± 11.4	53.17 ± 7.49
*A. muris*	0.028/0.52	−1.53/0.06	−0.21/0.39	−1.21/0.15	137.67 ± 35.22	28 ± 4.96	47.33 ± 34.43
*L. asaccharolyticus*	0.220/0.68	0.29/0.38	−0.86/0.18	−0.40/0.34	66.83 ± 5.79	63.67 ± 12.16	35.17 ± 5.21
*B. obeum A2–162*	0.166/0.64	0.15/0.45	0.60/0.29	0.93/0.18	49.67 ± 12.08	49 ± 17.88	53 ± 11.2

a Overall Kruskal–Wallis test expected *p*-value.

b Kruskal–Wallis expected Benjamini-Hochberg (eBH) corrected *p*-value.

c Effect values are CLR differences (negative sign indicates enrichment in treatment given the contrast ordering).

d Overlap quantifies distributional separation (0 = no overlap, 1 = complete overlap).

The following taxon-level findings for *L. sali*var*ius*, *L. phocaeense*, *L. crispatus*, *A. muris*, *F. plautii*, *B. hydrogenotrophica*, and *E. coli* are reported based on uncorrected Kruskal–Wallis/Wilcoxon *p*-values, alongside with ALDEx2 CLR effect sizes and distributional overlap. However, none remained significant after FDR correction (Table 1, all eBH ≥ 0.49). In addition, *L. crispatus* did not reach uncorrected significance (KW *p* = 0.087), and *E. coli* showed only borderline significance (KW *p* = 0.060). These results should therefore be interpreted as hypothesis-generating signals warranting validation in larger cohorts rather than statistically robust findings. While DAA did not reveal any differentially abundant taxa across the dataset, the Kruskal–Wallis and Wilcox pairwise test revealed taxa that potentially differ among treatment groups. For instance, *Ligilactobacillus salivarius* (3%) differed significantly among dietary treatments (KW, *p* = 0.035). Pairwise tests revealed significant difference of this taxa in Dulse (effect = −1.20, overlap = 0.08) and Soyabean meal (1.04/0.16) groups compared to the reference diet group (Wilcoxon, *p* < 0.05), supporting a robust enrichment of LAB/P taxa in both Dulse diet (408 ± 63.0) and Soyabean meal (408 ± 153) groups compared to the Reference diet (104 ± 32.8). By contrast, Dulse vs. Soyabean meal (effect = 0.32, overlap = 0.36) were nonsignificant, consistent with the high mean normalised counts observed in the Soyabean meal group, but much greater variability compared to the Dulse diet group. Similarly, while *Lactobacillus crispatus* (0.78%) showed a non-significant overall difference among dietary treatments (KW, *p* = 0.087), pairwise comparisons indicated a significantly higher effect and lower overlap (−1.36/0.08) in the Dulse group compared to Reference diet (Wilcoxon, *p* < 0.05), whereas Dulse vs. Soyabean meal were not significantly different, consistent with elevated mean counts of *L. crispatus* in Dulse-fed birds (189.33 ± 78.1), intermediate levels in the Soyabean meal (222.5 ± 126.28) and lower in reference diet (22 ± 3.87) groups. Albeit at low relative abundance, *Acutalibacter muris* (0.38%) also differed significantly among treatment (KW, *p* = 0.028); pairwise tests indicated significantly higher effect and lower overlap in both Dulse vs. Reference diet (−1.53/0.06) and Dulse vs. Soyabean meal (−1.21/0.15), indicating a relative enrichment in Dulse (137.7 ± 35.2) versus Soyabean meal (47.33 ± 34.43) and Reference diet (28.0 ± 5.0) treatment groups. In contrast, the reference diet exhibited relative enrichment of primary fermenters (SCFA producers) such as *Lachnoclostridium phocaeense* (1.85%), *Flavonifractor plautii* (1.22%), uncultured *Subdoligranulum* sp. (0.78%) and *Enterocloster bolteae* (0.34%). Among them, *L. phocaeense* differed significantly among dietary treatments (KW *p* = 0.036), with pairwise contrasts showing the largest CLR difference in the Dulse vs. Reference contrast (effect = 1.42, overlap = 0.10, Wilcoxon, *p* < 0.05), consistent with higher mean counts in the Reference diet (397.5 ± 65.5) compared to Dulse (368.83 ± 67.97) and Soyabean meal (266.0 ± 54.2). Likewise, *F. plautii* differed significantly across diets (KW, *p* = 0.009), with a higher effect and lower overlap in both Dulse vs. Reference diet (1.88/0.02, Wilcoxon, *p* < 0.05) and Reference diet vs. Soyabean meal (−0.89/0.13, Wilcoxon, *p* = 0.0524), consistent with higher mean counts in Reference diet (291.7 ± 63.2) compared to Dulse (226.33 ± 23.16) and Soyabean meal (162.5 ± 26.49) groups. Lastly, Soyabean diet exhibited relative enrichment of *Sellimonas intestinalis* (0.76%), *Blautia hydrogenotrophica* (0.52%) and *B. obeum A2–162* (0.27%) compared to the Dulse and Reference diet. Among them, *B. hydrogenotrophica* varied significantly across diets (KW, *p* = 0.020), and pairwise Dulse vs. Soyabean (effect = 1.26, overlap = 0.03) showed enrichment in Soyabean (127.0 ± 41.8), compared to both Reference diet (82.67 ± 15.66) and Dulse (78.33 ± 7.25) groups.

Taxa with pathogenic or opportunistic potential were also investigated due to their relevance in the poultry production industry (Supplementary Table S1). These included *E. coli*, *Clostridioides difficile*, *Enterococcus faecium*, *Klebsiella pneumoniae*, *S. enterica* and several *Clostridium*-related taxa (including *C. perfringens*, *C. botulinum*), which can act as opportunists or carry virulence/resistance determinants under dysbiotic conditions (Sharma S. et al., 2025; Sharma I. et al., 2025). Among them, *E. coli* (1% average relative abundance) varied across diets (KW, *p* = 0.0596), with pairwise contrasts showing the largest CLR difference in the Reference diet vs. Soyabean meal (effect = −1.42, overlap = 0.07, Wilcoxon, *p* < 0.05). *E. coli* contigs distribution was characterised by a higher mean count in Dulse samples (380.17 ± 176.88) but much greater variability, followed by Reference diet (146.67 ± 28.22) and Soyabean meal replicates (52.33 ± 9.28), indicating a diet-responsive enrichment of *E. coli* under the Dulse diet regimen. By comparison, *C. difficile* (0.45%), *E. faecium* (0.1615%) and *C. perfringens* (0.0675%) mean counts were found to be uniform across treatment groups.

### Antibiotic resistance genes, virulence factors and mobile genetic elements profile

To gain a comprehensive understanding of the mechanisms underpinning antimicrobial resistance, pathogenicity, and gene mobility within the chicken caecal bacteriome, resistome, virulome, and mobilome were profiled. After screening for the relevant genetic elements, a total of 921 long-reads representing 106 taxonomy groups were found to contain ARGs, VFs, MGEs, or a combination of these elements (Supplementary file 2). Among them, 557 long-reads contained ARGs (LR-ARG reads), corresponding to 80 distinct resistance genes spanning 17 antibiotic drug classes, 148 long-read reads contained VFs (LR-VFs reads), corresponding to 126 distinct virulence genes spanning 8 functional mechanisms, and 238 long-read reads contained genetic elements (LR-GEs reads), corresponding to 11 distinct plasmid replicons spanning 3 replicon families.

#### ARG profile across dietary treatments

**Figure 4:**
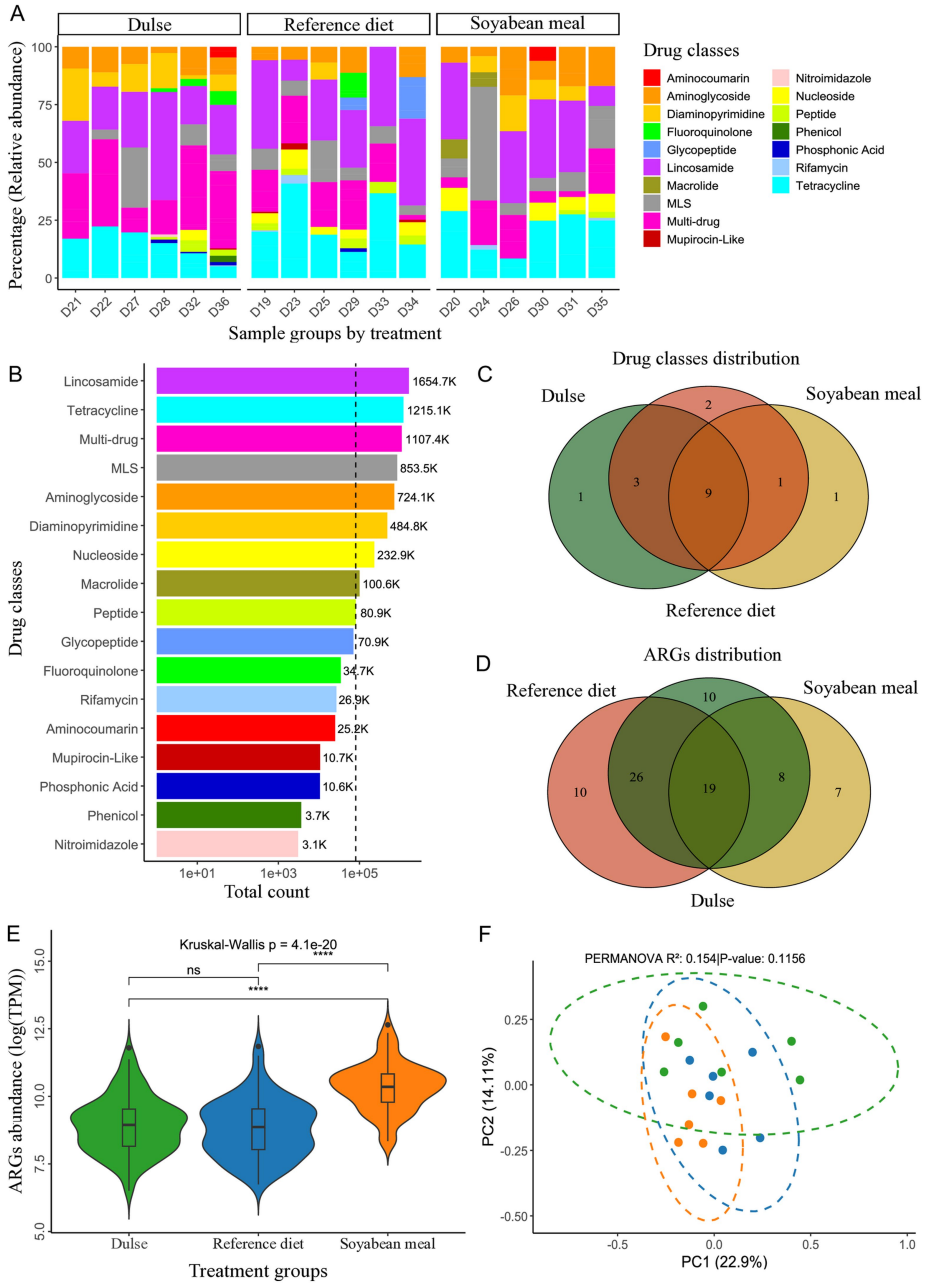
Diet effects on antibiotics resistance genes (ARG) across treatments. **(A)** Relative abundance of drug classes categories per sample grouped by treatment **(B)** Total counts (summed TPM) for each drug class across all samples. **(C)** Overlap of drug classes and ARGs **(D)** among treatments, values in each sector correspond to counts of unique/shared features. **(E)** ARGs abundance (log TPM) by treatment (Kruskal–Wallis global test). Boxplots are overlaid to show median and interquartile range. Pairwise comparisons are annotated above the plot; asterisks indicate significance levels (Wilcoxon *p* < 0.05). **(F)** Principal Coordinates Analysis (PCoA) of samples based on Bray–Curtis dissimilarities of ARG profile.

The ARG profile revealed a diverse array of resistance determinants across all chicken caecal samples (Supplementary file 2). The most frequently detected by read counts were *tet(W)* (102), *lnuC* (86), and *tet(O)* (43), highlighting the prevalence of tetracycline and lincosamide antibiotic class resistance within the caecal bacteriome. Additional ARGs included *erm(G)* (29), *erm(B)* (19), and *erm(52)* (16), which confer resistance to lincosamide, macrolide, and streptogramin antibiotics (MLS group) (Jia et al., 2017). The detection of *mef(En2)* (27), *APH(3′)-IIIa* (25), *dfrF* (18) and *SAT-4* (12) further confirmed resistance to macrolide, aminoglycoside, diaminopyrimidine and nucleoside antibiotics classes, respectively. Finally, genes such as *mdtE/F*, *cfrE* and *gadW/X*, associated with multidrug resistance and stress response regulation (Jia et al., 2017), were also present, though at lower frequencies (*n* < 10). The analysis of alpha diversity metrics showed that the total number of identified ARGs (richness), prevalence and abundance (Shannon diversity) were not significantly different (KW, *p* > 0.05) across the dietary treatments (Supplementary Figures S4A,B). Although the statistical tests show no significant difference, the Dulse treatment group (21.5 ± 5.17) exhibited the highest dissimilarity—especially in the D32 and D36 samples—in terms of richness and Shannon index values. In comparison, the Reference diet group (17.5 ± 1.91) and the Soyabean meal group (12.8 ± 0.792) demonstrated a more uniform richness among their respective samples. A total of 17 antibiotic classes were identified across all the treatment groups (Figure 4A), with 8 being the most abundant (*n* > 100 K, TPM) and prevalent (no. of samples > 10) among the caecal resistome profile (Figure 4B), suggesting a shared resistance profile among the caecal bacteriome. Analysis of shared ARGs between sample groups revealed that 26 ARGs conferring resistance to three classes were shared between Dulse and the Reference diet, while 8 ARGs across one class were shared between Soyabean meal and the Reference diet (Figures 4C,D). By comparison, 10 ARGs were unique to both the Dulse and Reference diets, while 7 were unique to the Soyabean diet. A total of 19 ARGs, conferring resistance to nine antibiotic classes (including lincosamide, tetracycline, multi-drug, MLS, aminoglycoside, diaminopyrimidine, nucleoside, macrolide, and peptide) were common across all sample groups (Figures 4C,D), underscoring a shared ARG profile of the caecal bacteriome. Kruskal–Wallis comparison between the groups revealed significant differences in ARG abundance among diets (KW, *p* = 0.01.6; Figure 4E). Similarly, pairwise comparisons showed that the Soyabean meal group exhibited significantly higher ARG levels than both the Reference diet and Dulse groups (W, *p* < 0.0001). In contrast, no significant difference was detected between the Reference diet and Dulse groups (W, *p* > 0.05). Collectively, the resistome of the GIT of poultry fed with the Soyabean meal diet showed lower richness and Shannon diversity yet higher ARG abundance values, which might indicate high abundance of a small group of ARGs in the caecal community on Soyabean meal fed birds.

**Figure 5:**
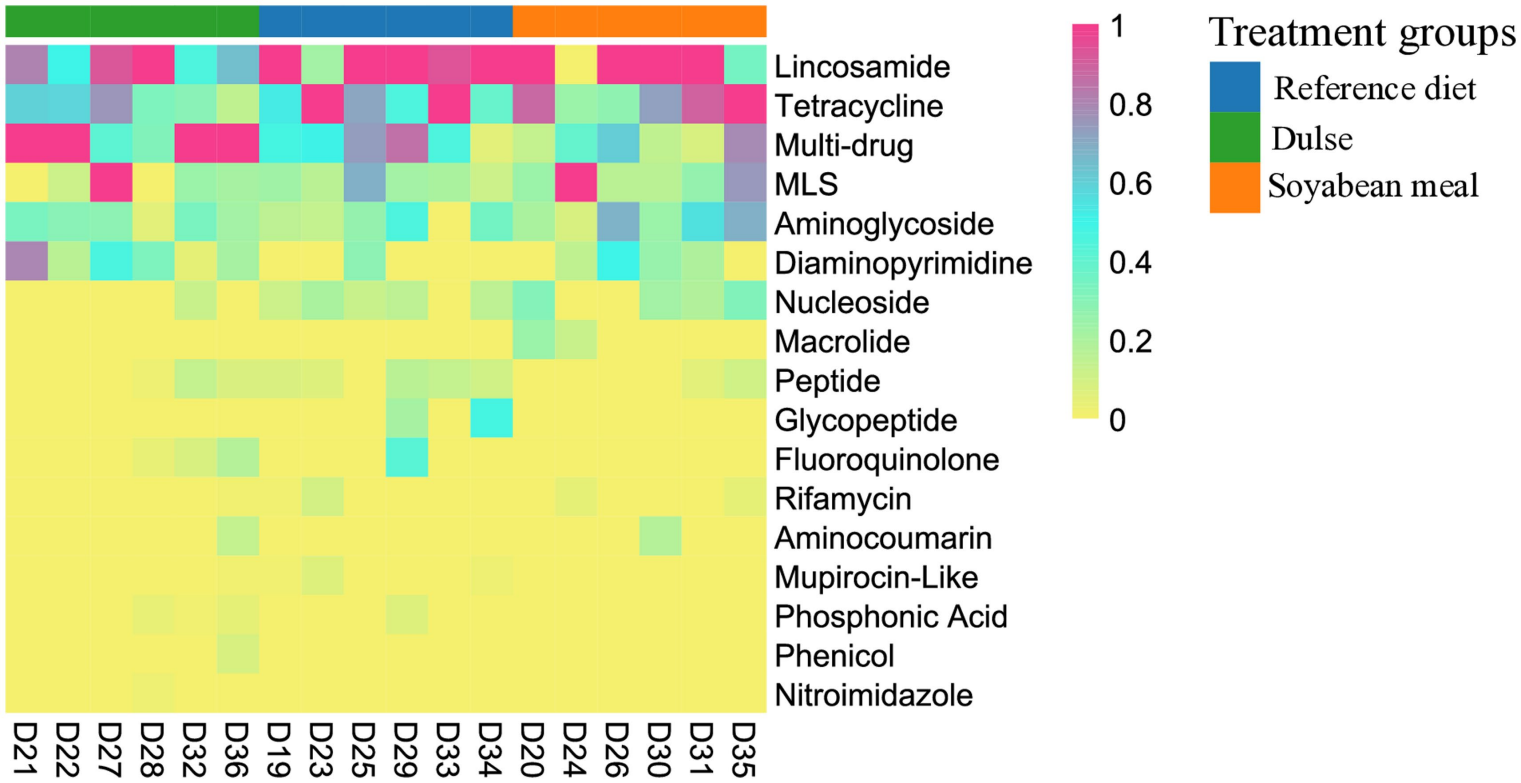
Heatmap of antibiotic classes abundance across dietary treatments. Each column represents a sample (D21–D36), and each row corresponds to a distinct ARG class. The colour intensity reflects normalised abundance values, ranging from 0 (low abundance) to 1 (high abundance), using a three-colour gradient: yellow (Low abundance), teal (Intermediate abundance), and Magenta (High abundance).

Beta-diversity based on Bray–Curtis dissimilarity scores was performed to assess the differences in ARGs composition between treatment groups. The results revealed a partial clustering of resistome profiles, with PC1 and PC2 explaining 22.9 and 14.1% of the total variation, respectively (Figure 4F). PERMANOVA analysis (adonis2, 9,999 permutations) indicated that dietary treatment accounted for 15.4% of the variation in resistome composition (*R*^2^ = 0.154), though the effect was non-significant (*p* = 0.116). This result suggests that while ARG abundance differed across treatment groups, the overall resistome composition was not significantly influenced by diet. Overall, six antibiotic classes (lincosamide, tetracycline, multi-drug, MLS, aminoglycoside, and diaminopyrimidine) had high relative abundance (0.6–1) in Dulse and Soyabean groups compared to the Reference diet (Figure 5), and fluoroquinolone, glycopeptide, and rifamycin exhibited lower relative abundance (0.2–0.6) in Dulse or Soyabean-fed groups. Among them, 11 high-abundance ARGs—spanning lincosamide (*lnuC*), tetracycline (*tet(W)*, *tet(O)*, *tet(40)*), diaminopyrimidine (*dfrF*), MLS (*ErmG*, *ErmB*, *Erm(52)*), macrolide (*Mef(En2)*), aminoglycoside (*APH(3′) − IIIa*), and nucleoside (*SAT−4*)—were prevalent across all treatment groups, with relative abundance values ranging from 0.6 to 1 (Supplementary Figure S5).

**Figure 6:**
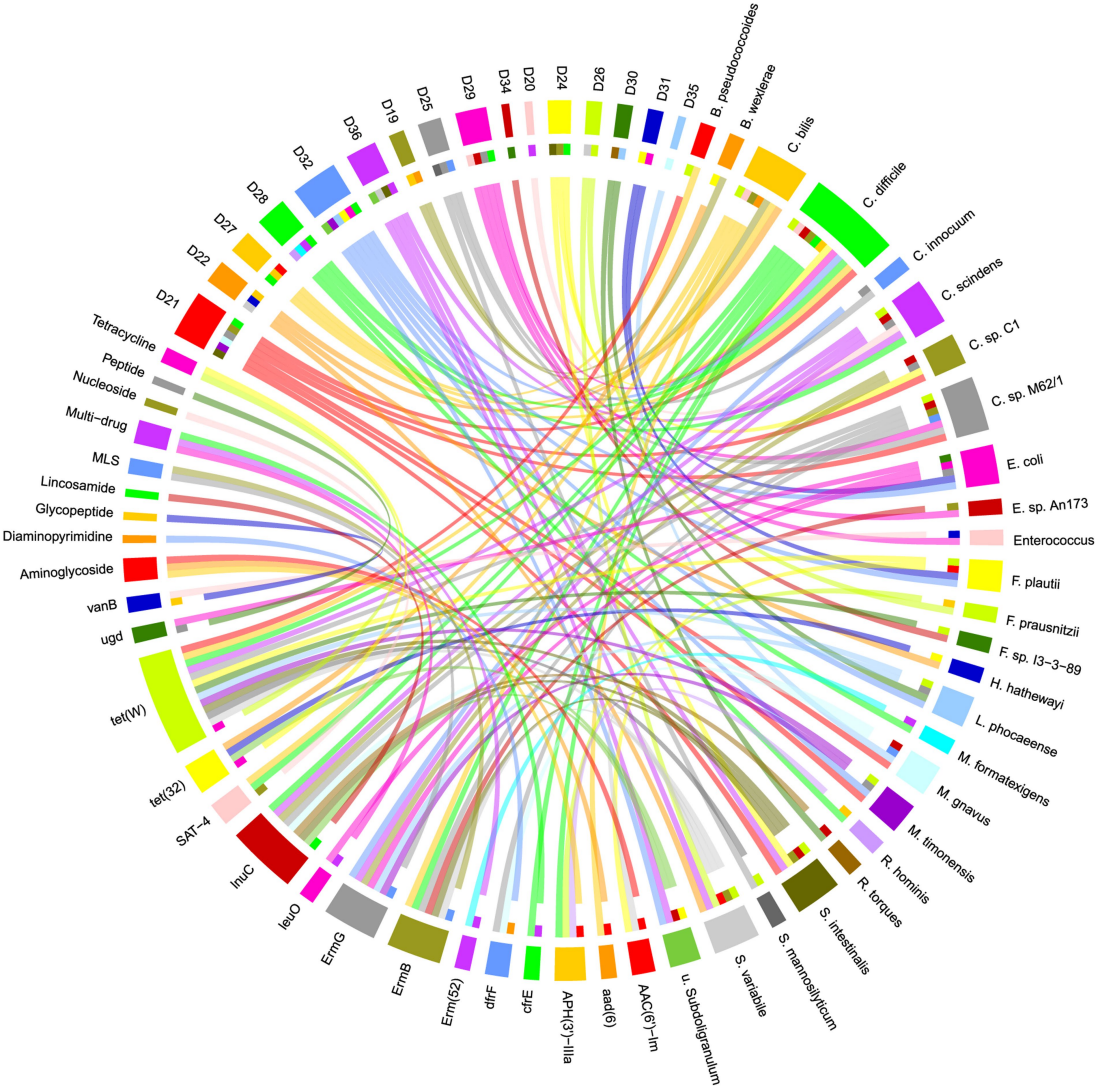
ARG associations between samples, microbial taxa, ARGs, and drug classes. Coloured ribbons connect ARGs to their corresponding drug classes and microbial hosts, revealing taxon-specific resistance profiles and potential reservoirs of resistance.

To investigate the resistant dynamics complexity of the chicken caecal bacteriome, resistome connectivity was carried out by linking the relationships among treatment groups, taxonomic groups, ARGs, and drug classes (Figure 6). The analysis revealed a complex interconnected nature of the resistome, with 15 ARGs linked to multiple bacterial taxa (*n* = 25), including pathogenic taxa (Supplementary Table S2). The complex connectivity across all sample groups reinforces the concept of a shared resilient resistome that persists regardless of dietary intervention. The gene *dfrF* was found to be enriched in Dulse (KW, *p* = 0.0269) and linked to reads classified as *Acutalibacter muris* (0.38%) described earlier (section 3.1.2). Similarly, *tet(32)* was also enriched in Dulse compared to the Reference diet and Soyabean meal (KW, *p* = 0.0518) and was associated with reads classified as *Subdoligranulum variabile* (0.83%), uncultured *Subdoligranulum* (0.78%), and *Hungatella hathewayi* (0.22%). In contrast, *tet(O)* was enriched in Soyabean meal and Reference diet compared to Dulse (KW, *p* = 0.0207) and associated with reads classified as *Lachnoclostridium phocaeense* (1.85%), *Massilistercora timonensis* (0.70%), *Claveliimonas bilis* (1.12%), *Ruminococcus gauvreauii* (1.12%) and *Dorea longicatena* (0.20%).

Other prevalent ARGs, such as *tet(W)*, *lnuC*, *APH(3′)-II*, and *ErmG* did not differ among diets (KW, *p* > 0.05) and were prevalent across most samples (*n* > 13). These ARGs presented a high connectivity to multiple species including highly abundant beneficial commensals such as *Lachnoclostridium phocaeense* (1.85%), *Massilistercora timonensis* (1.13%), and *Faecalibacterium prausnitzii* (1.20%). They were also connected to low abundant taxa with pathogenic potential such as *Clostridioides difficile* (0.45%), *Streptococcus suis* (0.01%), *Enterococcus faecium* (0.16%), and *Clostridium perfringens* (0.07%). Notably, *C. difficile* reads with multiple ARGs were persistent (0.45%, *n* = 1,523) among all treatment groups (section 3.1.2) (Supplementary Figure S6A,B).

#### Distribution of virulence factors across dietary treatments

**Figure 7:**
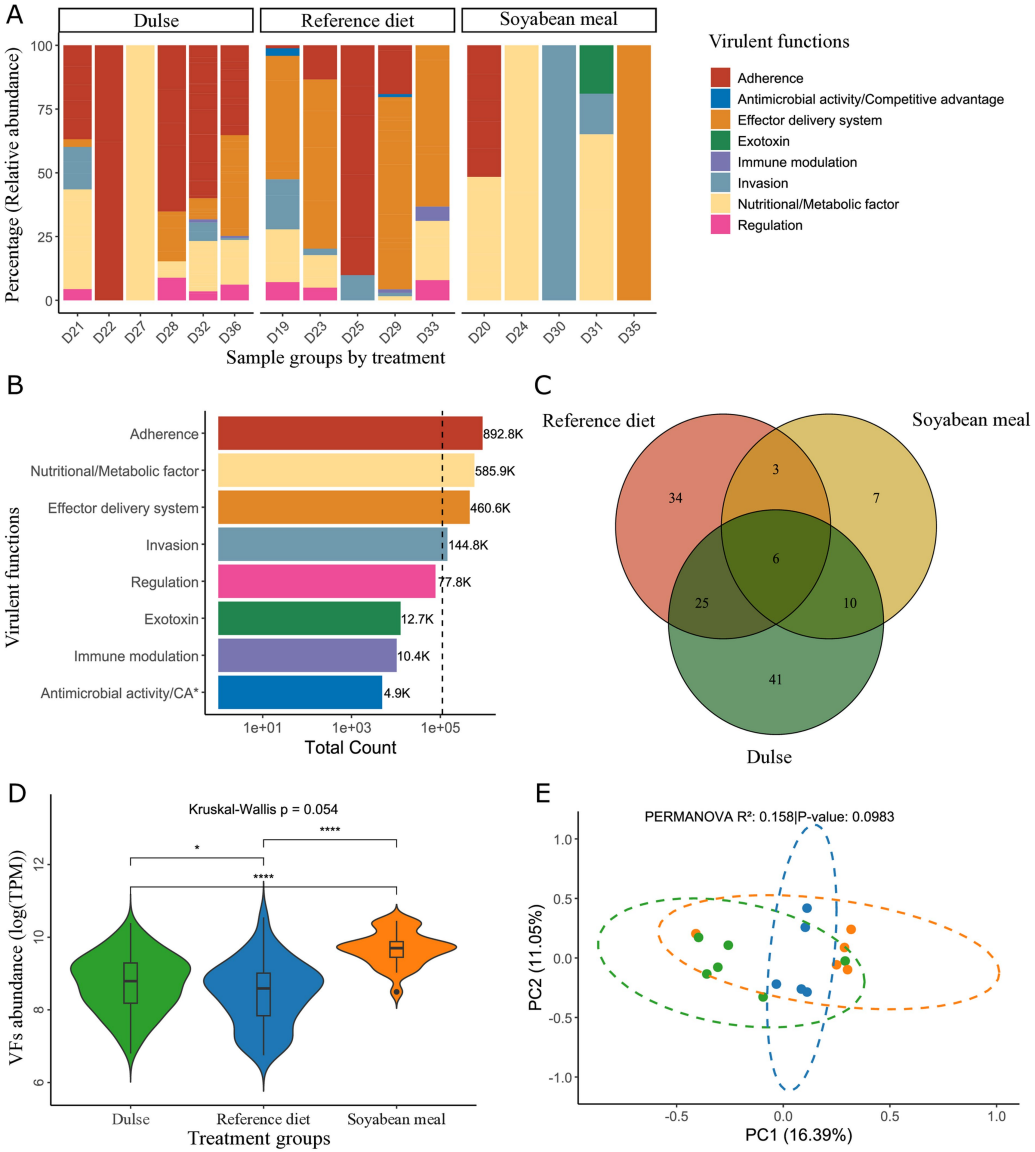
Diet effects on virulence factors across treatments. **(A)** Relative abundance of virulent function categories per sample grouped by treatment (Dulse, Reference diet, Soyabean meal). Colours indicate function categories. **(B)** Total counts (summed TPM) for each virulent function across all samples. Values shown on the *x*-axis are total TPM (thousands). **(C)** Overlap of virulent genes among treatments, values in each sector correspond to counts of unique/shared VFs between the indicated diet groups. **(D)** VFs abundance (log TPM) by treatment (Kruskal–Wallis global test). Boxplots are overlaid to show median and interquartile range. Pairwise comparisons are annotated above the plot; asterisks indicate significance levels (Wilcoxon, *p* < 0.05). **(E)** Principal Coordinates Analysis (PCoA) of samples based on Bray–Curtis dissimilarities of VFs profile.

The virulence factors (VFs) profile revealed a diverse array of virulent determinants across all chicken caecal samples (Supplementary file 2). The most frequently detected mechanisms were adherence (202), followed by effector delivery system (180), nutritional/metabolic factor (164), invasion (48), regulation (24), antimicrobial activity/competitive advantage (12), immune modulation (10) and exotoxin (2). Genes associated with adherence, effector delivery system and nutritional/metabolic factor were also the most abundant (*n* > 460 K, TPM) among the virulence profile of the caecal bacteriome (Figure 7B; Supplementary Figure S8). The comparison of relative abundances of these virulent mechanisms revealed a distinct profile across treatments (Figure 7A), with genes associated with adherence and effector delivery system mechanisms being enriched in Dulse and Reference diets compared to Soyabean meal (W, *p* < 0.001). By comparison, genes associated with Nutritional/Metabolic factor were significantly enriched in Soyabean meal (W, *p* < 0.0001).

Despite the observed differences in relative abundance in specific mechanisms, VFs richness revealed no statistically significant difference among treatments (KW, *p* = 0.0675), despite all Soyabean meal samples having values below the overall richness median (Supplementary Figure S7A). Similarly, the Shannon diversity was not significantly different between diet groups (KW, *p* = 0.0736) (Supplementary Figure S7B). Nonetheless, pairwise comparisons indicated a significant difference in Reference diet (15.6 ± 3.54) compared to the Soyabean meal (5.2 ± 2.40) group (Wilcoxon, *p* < 0.05), whereas other treatment pairs were not significantly distinct. The VFs distribution was very similar to that observed for ARGs, with a high variability in Dulse Group (24.5 ± 7.81), where samples D32 (richness of 52, Shannon index 3.9) and D36 (richness 42, Shannon index 3.7) showed substantially higher VFs richness/diversity than any other sample across all treatments.

A total of 25 VFs were shared between Dulse and the Reference diet group, 3 VFs were shared between Soyabean meal and the Reference diet, and 10 VFs between Dulse and Soyabean meal diets (Figure 7C). By comparison, a larger number of VFs were unique to each treatment (41 in Dulse, 34 in the Reference diet and 7 in Soyabean meal), while only 6 VFs were present across all treatment groups. Even though the overall Kruskal–Wallis test did not show significant differences in the dataset (KW, *p* = 0.054, Figure 7D), pairwise Wilcoxon comparisons revealed significantly higher VFs abundances in the Soyabean meal group compared to both the Reference diet and Dulse groups (W, *p* < 1 × 10^−4^), while the Dulse and Reference diet groups differed only marginally (W, *p* < 0.05). Beta diversity analysis based on Bray–Curtis dissimilarity distance matrix further revealed that dietary treatment explained 15.8% of the variation in virulence factor composition, while being non-significant (*R*^2^ = 0.158, *F* = 1.22, *p* = 0.098), indicating similar VFs distribution between treatment groups (Figure 7E). These compositional trends align with the observed abundance differences, particularly the elevated virulent functions observed specifically in each treatment group.

To investigate the virulent dynamics complexity of the chicken caecal bacteriome, virulome connectivity was carried out by linking taxonomic groups to virulent gene/functions among treatment groups (Supplementary Figure S9, S10). The analysis revealed a less interconnected virulome than the resistome, with 126 VFs linked to few taxonomy groups (*n* = 11), including pathogenic ones (Supplementary Table S2). Among all the caecal samples, *Enterobacteriaceae* family (1.533%) LR-VFs reads, [including *Escherichia* genus (0.1096%, *n* = 367) and *E. coli* (0.08%, *n* = 268)] were prevalent across 16 samples and related to 107 VFs. Among them, 26 VFs were involved in adherence functions encoding structural subunits, periplasmic chaperones, outer membrane ushers, and transcriptional regulators— for Type 1 fimbriae (*fim*), *E. coli* common pili (*yag/ecp*), and Colonisation Factor Antigen I (CFA/I) (*cfa* genes)—as well as specialised autotransporter adhesins (*fdeC*), which together enable bacterial adhesion, biofilm development, and environmental adaptation. A total of 53 VFs were involved in Effector delivery system (Type II, Type III, and Type VI secretion systems) were found, providing the essential machinery for assembling transmembrane conduits (*g*sp.*, esc, tss*) and delivering virulence factors (*nle, cif, eae*), allowing them to manipulate host cellular environments. Additionally, 29 VFs were involved in Nutritional/Metabolic factor encompassed biosynthesis genes (*ent*, *ybt*), membrane-specific receptors (*fepA*, *fyuA*), and intracellular release enzymes (*fes*)—for the enterobactin and yersiniabactin iron-acquisition systems, being vital for bacterial pathogenesis and metal homeostasis.

Furthermore, *Lachnoclostridium phocaeense* (1.16%, *n* = 3,885, Reference diet), and *Buchnera aphidicola* (0.01%, *n* = 34, *Dulse*) LR-VFs reads were linked to 11 VFs associated with Invasion mechanism including the *kps* operon (kpsC/D/E/F/S/U), which constructs a protective polysaccharide capsule for immune evasion, with a suite of specialised invasion factors (*ibeB/C*, *ompA*, and *aslA*) that facilitate bacterial attachment to and penetration of host cellular barriers; while 4 VFs associated with regulation mechanism which constitutes a core regulatory network, comprising the general stress sigma factor (*rpoS*) and specialised metal-sensing regulators (*pmrA*, *phoP*, *fur*), that coordinates bacterial adaptation to environmental stress, iron scarcity, and host-mediated antimicrobial attacks. In Soyabean meal, *Salmonella enterica* subsp. *enterica* serovar Typhimurium (0.001%, *n* = 6) LR-VFs contigs were linked to an enterotoxin *senB*. Notably, *Enterobacteriaceae* family (1.533%, *n* = 5,134) related LR-VFs reads differed significantly among treatment (KW, *p* = 0.01576), being particularly enriched in the Dulse group (593 ± 234), and Reference diet (291 ± 60.9) compared to Soyabean meal (106 ± 18.2) group, underscoring that this family members including *E. coli* possess a potential risk for increased virulence within the caecal microbial community (Supplementary Figures S11A,B).

### Mobile genetics elements profile

To investigate the potential for horizontal gene transfer and genomic plasticity within the chicken caecal bacteriome, we described the richness, diversity and relative abundance of the mobile genetic elements (MGEs) across all dietary treatments. The mobilome profile revealed a narrow array of mobile elements, with only *Col*-type (7), *IncF* (3) and *IncX* (1) being detected across all the chicken caecal samples (Supplementary file 2). Differences in MGEs richness and diversity between the diet groups were not statistically significant (KW *p* > 0.05) (Supplementary Figure S12A,B). Despite this, higher mean richness was observed in the Reference diet (4.33 ± 1.03) and the Dulse (4 ± 2.10) compared to the Soyabean meal (2.75 ± 0.5) group.

**Figure 8:**
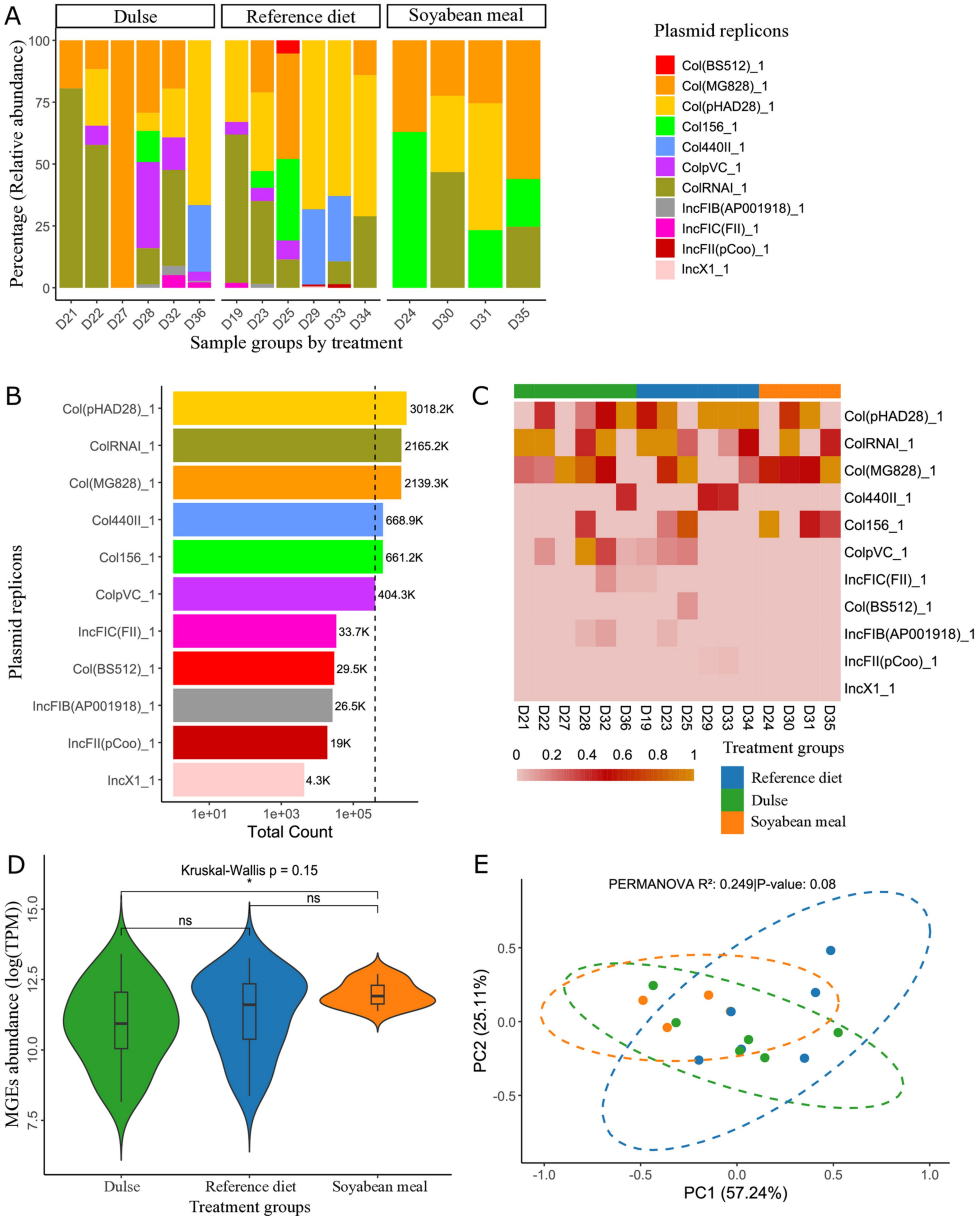
Diet effects on MGEs profile across treatments. **(A)** Relative abundance of plasmid replicons per sample grouped by treatment. **(B)** Total counts (summed TPM) for each plasmid replicons across all samples. Values shown on the *x*-axis are total TPM (thousands). **(C)** Heatmap of plasmid replicons abundance across dietary treatments. **(D)** MGEs abundance (log TPM) by treatment (Kruskal–Wallis global test). Boxplots are overlaid to show median and interquartile range. Pairwise comparisons are annotated above the plot; asterisks indicate significance levels (Wilcoxon, *p* < 0.05). **(E)** Principal Coordinates Analysis (PCoA) of samples based on Bray–Curtis dissimilarities of MGE profile.

A total of 11 plasmid replicons were detected across all the treatment groups (Figure 8A), with six Col-type types being the most abundant (*n* > 400 K, TPM). Among them, three Col-type types (*Col(MG828)_1*, *ColRNAI_1* and *Col(pHAD28)_1*) were the most prevalent (no. of samples = 11) among the mobilome profile (Figure 8B), with a relative abundance ranging from 0.4 to 1 (Figure 8C). The Kruskal–Wallis test revealed no significant differences in MGEs abundance among treatments (KW, *p* = 0.15; Figure 8D), while pairwise comparisons showed that the Dulse group and Reference diet exhibited significantly higher MGEs abundances than the Soyabean group, with only differences between Dulse and Soyabean meal treatment groups being significant (W, *p* < 0.05). Furthermore, the MGEs distribution measured as beta diversity based on Bray–Curtis dissimilarity distance matrix, revealed partial clustering of plasmid replicon by treatment group, with PC1 and PC2 explaining 57.24 and 25.11% of the total variation, respectively (Figure 8E). PERMANOVA analysis (adonis2, 9,999 permutations) indicated that dietary treatment accounted for 25% of the variation in mobilome composition (*R*^2^ = 0.249), although the effect did not reach statistical significance (*p* = 0.08), indicating that the microbial communities were similar regarding their mobilome.

The mobilome connectivity further revealed a narrower interconnected nature compared to the resistome profile, with only 11 plasmid replicons linked to 10 taxonomy groups, including pathogenic ones (Supplementary file 2). For instance, *Enterobacterales* (1.9525%, *n* = 6,539) reads were associated with a few plasmid replicons (*ColRNAI_1*, *Col(pHAD28)_1*, *ColpVC_1*, *Col(MG828)_1* and others) in Dulse samples, revealing a tightly connected mobilome. Interestingly, *ColpVC_1* plasmid replicon was enriched in Dulse (KW *p* = 0.04) and linked to *E. coli* contigs (0.08%) described earlier (section 3.1.2). Additionally, *E. coli* reads were related to *Inc-type* replicons (*IncFIB(AP001918)_1*, *IncFIC(FII)_1*) that are known conjugative backbones linked to antimicrobial resistance dissemination. *Gammaproteobacteria* (2.696%, *n* = 9,029) and *Pseudomonadota* (3.9752%, *n* = 13,313) reads overlapped on *Col(MG828)_1*, *Col(pHAD28)_1* and *Col440II_1*, suggesting cross-taxonomic sharing of small *Col-type* plasmids within specific microbial community members.

In the reference diet samples, the mobilome was also centred on the same small *Col*-type plasmid replicon seen in Dulse, but with a subtly different connectivity pattern. For example, a greater representation of *Inc*-type replicons within *Enterobacteriaceae* (1.533%) reads (notably *IncFIC(FII)_1*, *IncFII(pCoo)* and *IncX1*) and an instance of *Col(BS512)_1* was linked to *Enterobacter ludwigii*. Meanwhile, *E. coli* contigs (0.08%) in Reference samples were also associated with *Col(MG828)_1*, *ColpVC_1* and *Col(pHAD28)_1* but lacked the *IncFIB*/*IncFIC* signatures observed in *E. coli* from Dulse-fed groups. Similarly, in the Soyabean meal group, the top three *Col* type plasmid replicons were also related to *Enterobacteriaceae* (1.533%) reads, which included several taxa with pathogenic potential, such as *E. coli* (0.08%), *K. pneumoniae* (0.01%), and *S. enterica* subsp*. enterica* serovar Typhimurium (0.001%). Overall, the mobilome was dominated by the top three plasmid replicons—*ColRNAI_1*, *Col(MG828)_1* and *Col(pHAD28)_1* mediating horizontal gene transfer among *Enterobacterales* and related taxa in the caecal ecosystem. The presence of short, high copy *Col*-type elements and larger conjugative *IncF*-type plasmid replicon in *E. coli* raises a particular concern because of their association with multidrug resistance gene carriage.

### Microbial community connectivity to genetic elements

**Figure 9:**
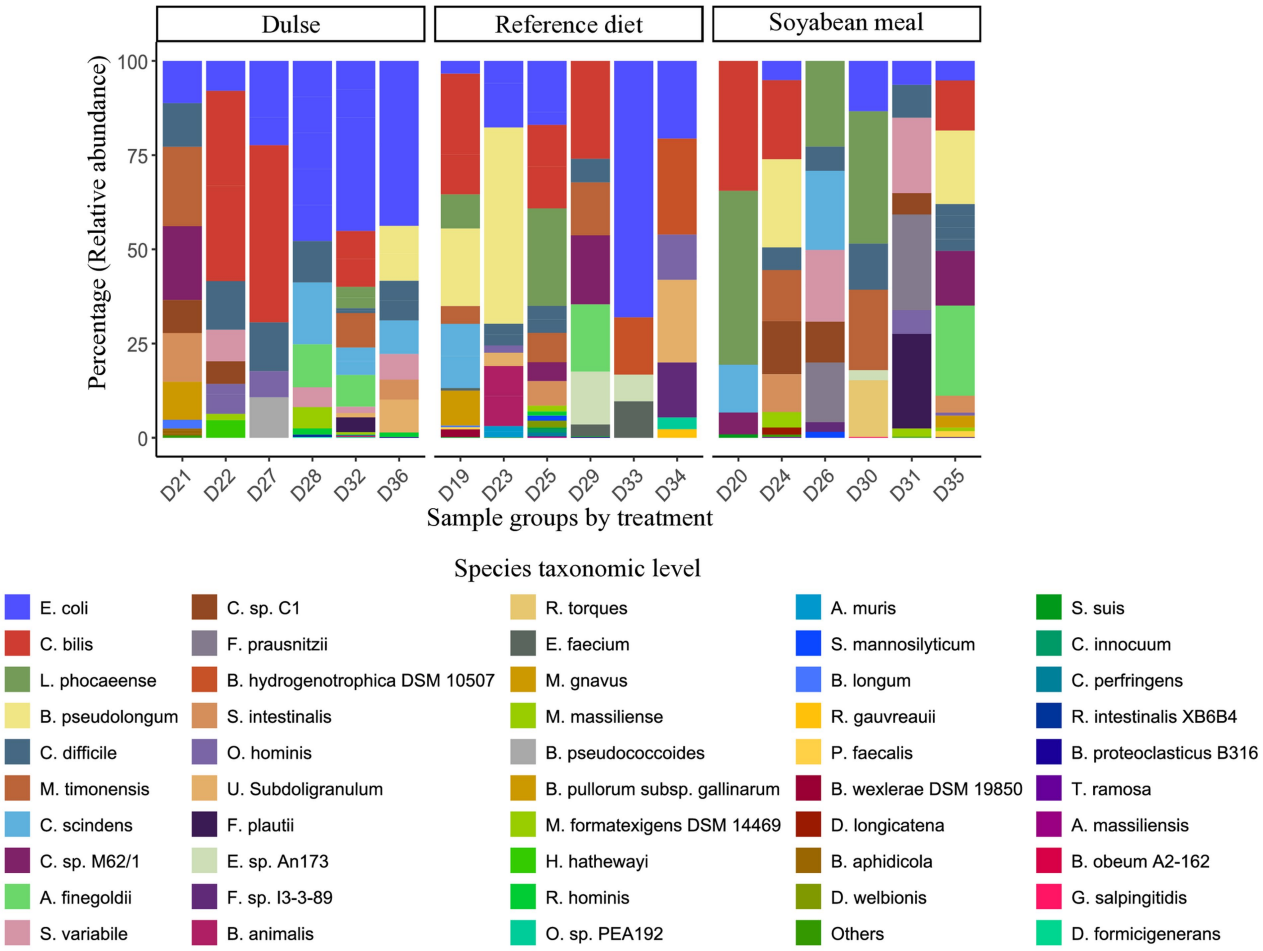
The figure illustrates the top 50 most abundant taxa carrying genetic elements across all chicken caecal samples; the *y*-axis represents the relative abundance at the species level in descending order, and the *x*-axis represents the sample groups by treatment.

The potential risk of resistance and virulence traits transmission within the chicken caecal bacteriome was further evaluated. Overall, 75 distinct related taxa at the species level were related to unique reads carrying genetic elements (GEs), including 29 ARGs, 25 VFs, and 9 MGEs. Among them, 12 taxa were present in all treatment diets, while a larger number of taxa were unique to each diet group (17 Reference diet, 17 Dulse, and 13 Soyabean meal). However, most of these taxa were associated with ARGs 89.33% (*n* = 67) alone, and only 4% (*n* = 3) were related to ARGs, VFs, and/or MGEs simultaneously (Supplementary Figure S13A,B), with a small set of high abundance taxa, most notably *E. coli*, *C. bilis*, *L. phocaense*, *B. pseudolongum, and C. difficile* (Figure 9). These dominant carriers were prevalent across all treatments (section 3.1.2), indicating a shared set of GEs hosts, but their abundance differed by diet and sampling point, revealing treatment and time-dependent modulation of GEs load rather than wholesale community replacement.

**Figure 10:**
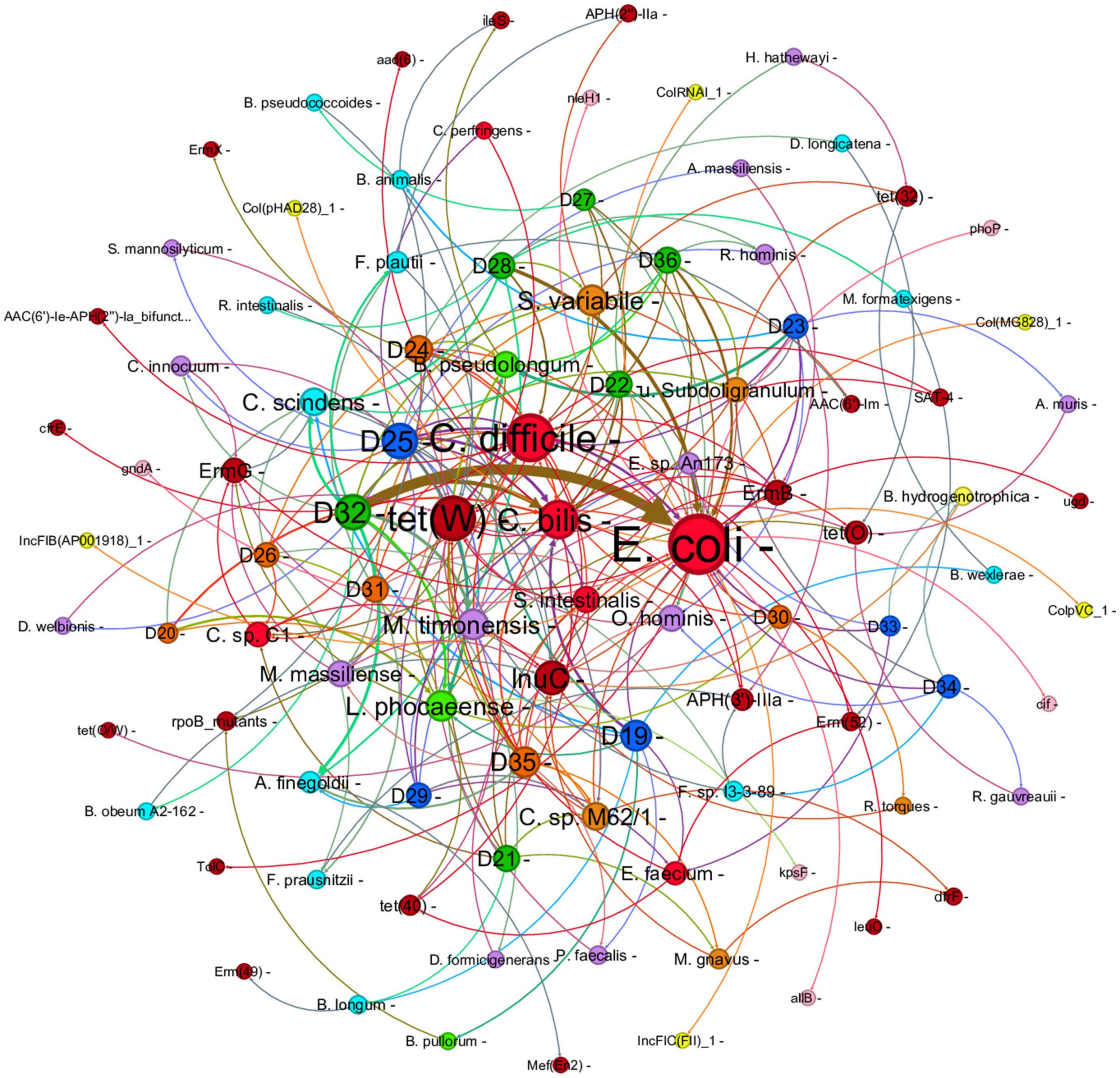
Network visualisation of the chicken caecal bacteriome composition under diet conditions. The nodes were distributed using the Fruchterman-Reingold force-directed layout algorithm in Gephi (v. 0.9.2). The network comprises a bipartite graph with various nodes representing experimental groups (Dulse, Reference diet and Soyabean treatment groups), bacterial taxa (functional groups), and genetic elements (ARGs, VFs, and MGEs), coloured by categories. Edges indicate a direct association or presence (e.g., a sample contains a specific taxon, or a taxon contains a specific gene). The thickness of edges can reflect abundance (taxa) and/or binary presence (GEs).

**Figure 11:**
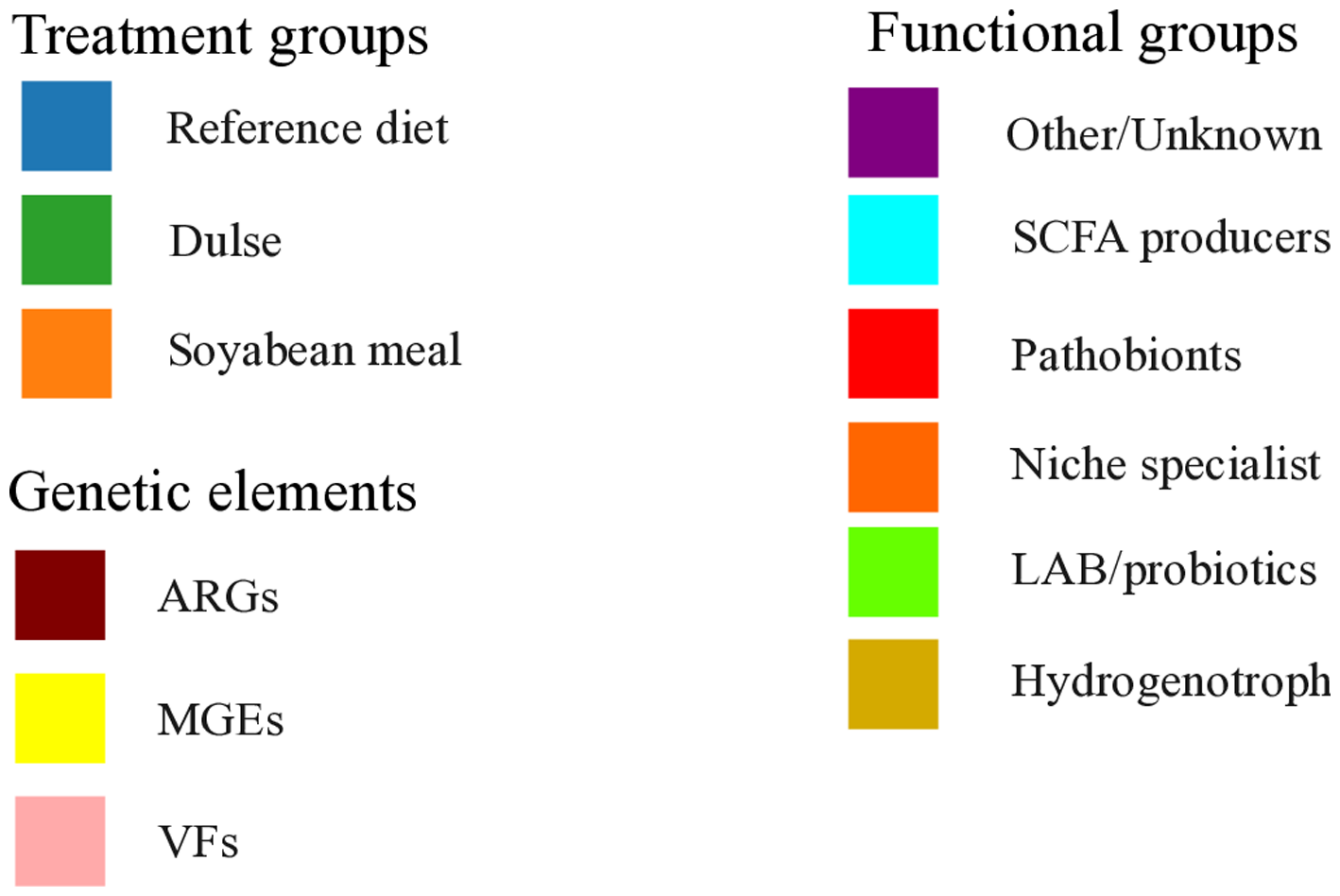
Colour key for the taxon–gene co-occurrence network shown in Figure 10, indicating treatment groups, genetic element types (ARGs, MGEs, VFs), and bacterial functional/taxonomic groups.

Network reconstruction revealed *E. coli* as the highest-degree node (degree *k* = 32, betweenness centrality = 0.30), connected to 17 distinct genetic elements: 5 ARGs (*TolC*, *CTX-M-101*, *ugd*, *leuO*, *ErmG*), 7 MGEs (predominantly small *Col*-type and *IncF* plasmid replicons), and 5 VFs (*allB*, *gndA*, *phoP*, *cif*, *nleH1*). The opportunistic *C. difficile* (*k* = 22, betweenness = 0.14) and *Claveliimonas bilis* (*k* = 16, betweenness = 0.13) were the next-most connected taxa, linked to 8 and 7 ARGs respectively, spanning tetracyclines (*tet(W)*, *tet(O)*, *tet(40)*), MLS (*ErmB*, *ErmG*, *Erm(52)*), aminoglycosides (*APH(3′)-IIIa*, *aad(6)*), nucleoside (*SAT-4*), and oxazolidinone-phenicol *cfrE* (in *C. difficile* only)—consistent with a multidrug carriage profile. At the gene level, *tet(W)* was the most broadly distributed ARG across taxa (*k* = 19), followed by *lnuC* (*k* = 12), *ErmG* (*k* = 7), and *ErmB* (*k* = 7), identifying these elements as core members of the resilient caecal resistome. Because taxon-GE pairs in this network were defined by direct read-level linkage (ABRicate screening of taxonomically classified reads; see Methods), the high centrality of *E. coli*, *C. difficile*, and *C. bilis* positions them as principal candidate carriers of these elements within the caecal ecosystem, and as potential intermediaries for co-selection under varying ecological pressures, including antibiotics or functional feed additives (Figure 10; colour key in Figure 11). While beneficial primary fermenters and mucin/glycan degraders—such as *F. prausnitzii* (*k* = 3, betweenness = 0.01) and *S. variabile* (*k* = 11, betweenness = 0.085) (enriched in the Reference diet; section 3.1.2) occupied more isolated ecological niches with significantly lower GE carriage, suggesting that while horizontal gene transfer is prevalent among opportunistic “hubs,” commensals remain relatively isolated from the antibiotic resistance burden within the caecal ecosystem (Figure 10).

Notably, the high prevalence of the *Col(MG828)_1* plasmid replicon, along with ARG, indicates potential mobility across caecal bacterial populations, thereby facilitating the dissemination of resistant/virulent traits. Worryingly, in SCFA-producer *L. phocaeense* (*k* = 11, betweenness = 0.14), VFs *kpsF* (involved in capsular polysaccharide biosynthesis, potentially enhancing immune evasion) co-existed with multiple tetracyclines (*tet(W)*, *tet(O)*, *tet(40)*) and macrolide (*ErmG*) resistance genes, respectively, raising concerns about the potential for pathogenicity traits to be co-transferred with resistant genes among beneficial GIT microbial populations.

## Discussion

The poultry industry faces increasing regulatory and societal pressure to reduce the use of antibiotics, owing to their contribution to the selection and dissemination of multidrug-resistant bacteria, with direct implications for animal health, public health and the long-term sustainability of production systems (Hedman et al., 2020; Zhou et al., 2020; Sana et al., 2025). As a result, dietary interventions that promote gut health and resilience without reliance on antibiotics have gained attention (Sharma S. et al., 2025; Sharma I. et al., 2025). Marine macroalgae such as *Palmaria palmata* (dulse), offer promise as environmentally sustainable feed ingredients, as they are rich in polysaccharides, polyphenols, and other bioactive compounds with the potential to modulate the gut microbiota and support host immunity (Khattak et al., 2025; Shannon et al., 2021). However, the microbial and genetic responses of the poultry gut microbiota to dulse supplementation remain poorly characterised. Recent studies have used advanced sequencing technologies to gain a deeper understanding of poultry gut communities, showing how metatranscriptomics and long-read metagenomics can reveal antibiotic-driven shifts in resistance gene expression and recover previously uncharacterised microbial genomes (Koorakula et al., 2022; Zhang Y. et al., 2022; Zhang T. et al., 2022). In this study, we used long-read metagenomics to investigate the impact of dietary dulse inclusion on the caecal bacteriome and its associated genetic repertoire across three dietary treatments. Importantly, the 30% dulse inclusion level used in this study represents a high experimental level employed as a sensitivity model to maximise detection of potential microbiome responses, and exceeds typical commercial inclusion rates used in poultry diets.

Our findings revealed no statistically significant effect of Dulse and Soyabean supplemented diets on the overall microbial composition of the poultry GIT microbiota. Nonetherless, birds receiving the Dulse supplemented diet consistently showed higher mean relative abundance values and greater variability of taxa, particularly in samples D32 and D36 (section 3.1 and 3.2). Similarly, most pairwise comparisons were largely non-significant, with the exception of VFs richness, for which the Reference diet group differed significantly from the Soyabean meal diet group (Wilcoxon, *p* < 0.05), suggesting potential modulation of virulent gene diversity driven by diet. These localised shifts may be driven by dulse-derived polysaccharides and bioactive compounds selectively promoting low-abundance taxa (Yadav and Jha, 2019), or may reflect microbial signals introduced from the dulse’s own host-specific microbiome (Corr et al., 2025). Future studies should therefore consider this variability and characterise the microbiome of dulse supplemented feed directly, in order to determine its contribution as a potential source of microbial taxa or microbial-derived signals influencing the poultry GIT microbiome.

Overall, the microbial profile across samples revealed a clear predominance of the class *Clostridia*, accounting for approximately 71% of the total reads across all dietary groups (Figures 1A,B), reflecting a mature and metabolically stable gut ecosystem (Lopetuso et al., 2013). Follow by the class *Bacilli* represented approximately 8% and included the genus *Lactobacillus*, recognised for maintaining caecal barrier integrity and competitive exclusion of pathogens (Yadav et al., 2017). The presence of *Bacteroidia* (6%), *Gammaproteobacteria* (5%), and *Actinomycetes* (5.9%) across samples further indicates a balanced gut microbial community associated with immune modulation and antimicrobial metabolite production (Kogut, 2019; Chen et al., 2022). At species level, the GIT microbiome was dominated by four functional groups: (1) Primary fermenters/SCFA producers, central to fibre degradation and butyrate/propionate production (Aruwa et al., 2021); (2) Lactic acid bacteria/probiotics (LAB/P), associated with carbohydrate fermentation, pH modulation and pathogen exclusion (Montso et al., 2022); (3) Proteolytic/opportunistic taxa and pathobionts; and (4) Mucin/glycan degraders. Among them, *A. finegoldii* (4%) ferments complex polysaccharides into SCFAs, particularly propionate, contributing to gut health by lowering intestinal pH and enhancing mucosal immunity (Shin et al., 2023). Secondary bile producer *C. scindens* (1.74%) plays a critical role in inhibiting enteric pathogens such as *Clostridium difficile* and *Salmonella* (Greathouse et al., 2015). LAB/P such as *L. sali*var*ius* has been consistently associated with improved growth performance and enhanced gut barrier function, supporting its potential as a probiotic intervention in antibiotic-free poultry production systems (van Zyl et al., 2020).

When interpreting the differential abundance patterns identified in this study, it is critical to view these taxon-level shifts as exploratory, hypothesis-generating signals rather than statistically established findings (see section 3.1.2), as none of these features remained significant after applying stringent empirical Bayes Benjamini-Hochberg correction. Our interpretations rely on uncorrected non-parametric trends and compositional effect sizes, meaning these observations require strict validation in a larger cohort before definitive conclusions can be drawn. Nevertheless, they offer valuable insights into how distinct dietary substrates shape the caecal ecosystem, revealing contrasting shifts in short-chain fatty acid producers, lactic acid bacteria, and potential pathobionts. For instance, taxa associated with SCFA production (*Lachnoclostridium phocaeense* (1.85%), *Flavonifractor plautii* (1.22%), an uncultured *Subdoligranulum* sp. (0.78%) and *Enterocloster bolteae* (0.34%)) were enriched in birds receiving the Reference diet (section 3.1.2). In contrast, LAB/P (*Ligilactobacillus salivarius* (3%), *Lactobacillus crispatus* (0.78%)) were enriched in the Dulse diet group. The combination of a large effect size (1.2) and minimal distributional overlap (0.08) for Dulse vs. Reference supports a robust enrichment of *L. salivarius* in the Dulse diet, likely via increased availability of fermentable carbohydrates (Montso et al., 2022). Most proteolytic/opportunistic taxa, including recognised pathobionts (*E. coli*, *Clostridioides difficile*, *Enterococcus faecium*, *Klebsiella pneumoniae*, *S. enterica*) and several *Clostridium*-related taxa (including *C. perfringens*, *C. botulinum*), did not differ significantly among dietary treatments (KW, *p* > 0.5); except *E. coli* (1%), which was significantly enriched in birds fed with Dulse and Reference diets and reduced under Soyabean meal (section 3.1.2). Although most *E. coli* strains are commensal, particular pathotypes such as avian pathogenic *E. coli* (APEC) are associated with colibacillosis and economic losses in poultry (Mehat et al., 2021; Azam et al., 2020). The presence of *C. difficile*, a producer of potent toxins (TcdA and TcdB) (Kordus et al., 2022) with colonisation rates up to 62% in healthy poultry (Weese, 2020), and *C. perfringens*, a causative agent of necrotic enteritis (Fathima et al., 2022), warrants futher monitoring despite their low abundance (Rychlik, 2020). Mucin/glycan degraders (*R. torques* (0.71%); *M. gnavus* (0.61%); *S. variabile* (0.83%)) showed no differential abundance among dietary treatments (KW, *p* > 0.5). In contrast, *Blautia hydrogenotrophica* (0.52%), a genus with reported functional and probiotic properties (Liu et al., 2021), was significantly enriched in Soyabean (KW, *p* = 0.020). In general, key beneficial taxa responded positively to all experimental diets, contributing to potential improvements in caecal functional capacity and nutrient utilisation while maintaining low relative abundance of putative pathogenic taxa.

Dietary treatment had a measurable impact on the abundance of genetic determinants. The Soyabean meal group showed significantly higher abundances of ARGs and VFs (W, *p* < 0.0001) despite exhibiting lower richness and diversity, indicating a community dominated by a few highly abundant resistant and virulent genes. By contrast, the composition of MGEs was nonsignificant across all dietary treatments. Notably, a highly interconnected resistome was revealed, with 15 ARGs linked to 25 bacterial taxa, encompassing both commensal and potentially pathogenic lineages (section 3.2.1). This observed stability may reflect long-term selective pressures, including historical antibiotic exposure or vertical transmission within the caecal bacteriome (Li et al., 2022; Juricova et al., 2021). Notably, *C. difficile* (0.45%, *n* = 1,523) reads were associated with a broad repertoire of ARGs, including *APH(3′)-IIIa*, *cfrC*, *Erm(B)*, Erm(42), *hucC*, *Sat4-A*, and *tet(M)*, suggesting it may act as a stable reservoir for antimicrobial resistance despite its low abundance. Additionally, sequencing data revealed that *Enterococcus* spp. reads, including those classified as *E. faecium*, were associated with *vanB* gene sequences, suggesting that poultry GIT microbiomes may harbour genetic determinants associated with vancomycin resistance (Yushchuk et al., 2020; Manyi-Loh et al., 2018). The persistence of this resistome despite dietary modulation with dulse indicates that the caecal microbiota maintained functional stability, while specific members may continue to act as reservoirs of antimicrobial resistance.

In contrast, the virulome showed a more constrained connectivity pattern, predominantly represented by reads of the *Enterobacteriaceae* family, including genus *Escherichia* and *E. coli*, detected across 16 samples and linked to 107 virulent genes (section 3.2.2). Members of the *Enterobacteriaceae* were primarily associated with adherence mechanisms involved in initial colonisation (Stones and Krachler, 2016), effector delivery systems (Type II, III, and VI secretion systems) mediating host-pathogen interactions (Slater et al., 2018; Bleves et al., 2020; Shaliutina-Loginova et al., 2023), and iron-acquisition systems (enterobactin and yersiniabactin) enhancing bacterial survival and gut colonisation (Ellermann and Arthur, 2017; Amiri et al., 2025; Diamant et al., 2024). In the reference diet, *L. phocaeense* and *B. aphidicola* reads were associated with virulence factors implicated in immune evasion, host cell invasion, and stress adaptation (Mao et al., 2025). Key global regulators identified included *phoP*, *rpoS*, *pmrA*, and *fur*, which modulate expression of virulence traits in response to environmental stressors (Mao et al., 2025). Reads attributed to *Salmonella enterica* serovar typhimurium in the Soyabean meal group harbored the enterotoxin gene *senB*, highlighting virulent functions driven by specific diet (Rychlik and Barrow, 2005). Although members of the *Enterobacteriaceae* family were detected across all dietary groups, their relative abundance remained low, consistent with typical profiles of a healthy poultry caecal microbiome (Plata et al., 2022; Koorakula et al., 2022; Zhang Y. et al., 2022; Zhang T. et al., 2022). Notably, the Dulse and Reference diets showed a modest enrichment of *Enterobacteriaceae* compared with the Soyabean meal group (KW, *p* = 0.01576), suggesting that the Soyabean diet may exert a stronger suppressive effect on opportunistic *Enterobacteriaceae* members, particularly *E. coli* (Supplementary Figure S13A,B).

Mobilome revealed a relatively compact yet functionally significant network, comprising 11 plasmid replicons associated with 10 taxonomic groups, including pathogenic taxa (section 3.2.3). Among these, only *Col*-type (7), *IncF* (*n* = 3) and *IncX* (*n* = 1) plasmids were consistently detected across all chicken caecal samples (Supplementary file 4). Previous studies have reported that high prevalence of short, high copy *Col*-type plasmids in enteric bacteria such as *E. coli* and *Salmonella* possesses a significant role in the maintenance and horizontal transfer of virulent and resistant determinants within the gut environment (Mellor et al., 2022). In contrast, the *IncF* plasmid replicon family is well-documented for its role in the spread of multidrug resistance (MDR) and virulence, harboring genes encoding extended-spectrum *β*-lactamases (ESBLs), adhesins, and toxins (Pitout and Chen, 2023). Interestingly, both *Col*-type plasmids and *Inc*-type plasmid replicons were incremented in the Dulse group (W, *p* < 0.05) and were predominantly associated with members of the *Enterobacteriaceae* family, including the pathogens *Escherichia coli*, *Escherichia albertii, Klebsiella pneumoniae*, and *Salmonella enterica* (section 3.2.3), reflecting microbial adaptability with potential implications for horizontal gene flow and dissemination of antimicrobial resistance (Branger et al., 2019; Foley et al., 2021). Importantly, these findings indicate that dulse inclusion was biologically active and associated with selective modulation of the poultry gut mobilome. Although specific plasmids (e.g., *ColpVC_1* and *IncF* types) were enriched in the dulse-fed group, no evidence was observed for widespread disruption of the overall genetic architecture. This suggests that dulse may exert targeted effects on mobilome composition without broadly disrupting its structure, supporting its potential as an alternative and/or supplementary feed ingredient. Nevertheless, given the substantial reservoir of resistome and virulome elements in the chicken caecal microbiome, these mobile genetic elements may act as conduits for the transfer of resistance genes through the food chain or environmental routes, including soil and water contamination via faecal waste, thereby impacting both animal and human health (Wang et al., 2024).

Microbial network analysis further revealed high connectivity among dietary treatment groups, bacterial taxa, and genetic elements (section 3.3). A subset of opportunistic taxa emerged as central nodes, with their connectivity strongly associated with multiple ARGs, including *tet(W)*, *lnuC*, and *ErmB* across all treatment groups. Adjacent MGEs (plasmid replicons) in the network suggest plausible mobilisation pathways that may transfer multidrug cassettes to other caecal residents. In contrast, primary fermenters enriched in the Reference diet and mucin/glycan degrading taxa were positioned as distant nodes with reduced genetic element cargo, consistent with previous reports (Dongre et al., 2025). Notably, *E. coli*, *C. difficile* and *C. bilis* associated reads emerged as key reservoirs of both resistance and virulence traits (Navarro et al., 2018; Zhang Y. et al., 2022; Zhang T. et al., 2022; Al-Zahrani, 2023). Despite these associations, only 4% of the total taxa concurrently harbored genetic elements, indicating that such high-risk genetic configurations are relatively rare. The persistence of beneficial taxa further implies a compensatory microbial equilibrium supporting intestinal functionality, even under this high experimental dulse inclusion level (Shin et al., 2023; van Zyl et al., 2020).

The potential of dulse lies not just in its nutritional value but in its ability to foster ecosystem resilience; by promoting a diverse, niche-stabilised microbiota, it minimises the proliferation of opportunistic pathogens, thereby lowering the probability of horizontal gene transfer and co-selection (de Jesus Raposo et al., 2016; González-Ortiz et al., 2019). Previous studies have shown that dulse bioactive compounds, including phenolics, carotenoids, and phycobiliproteins, exhibit antioxidant and mild antimicrobial properties that can enhance intestinal redox balance and modulate immune responses (Gupta and Abu-Ghannam, 2011; Moroney et al., 2013; Shannon et al., 2021). Dietary inclusion of dulse did not significantly alter the GIT bacteriome, neither destabilised microbial communities by promoting taxa with pathogenic potential nor increased the abundance of AMR and virulence associated genes. The functional plasticity observed in our study is consistent with adaptive metabolic reprogramming in response to the complex matrix of fermentable xylans, minerals, and bioactive compounds present in dulse, though the contribution of individual components remains to be determined. These results support dulse as a safe and efficacious feed ingredient in poultry production. From a circular sustainability perspective, dulse is a low-input marine resource; unlike synthetic additives that can persist in the environment, its bioactive glycans are biodegradable, preventing the cycle of resistance selection that often accompanies traditional poultry production.

While the 30% inclusion level used in this study exceeds those in commercial poultry feed formulations, it provides a sensitive model to evaluate the adaptive capacity of the gut microbiome to dulse-derived substrates. The preservation of a stable bacteriome and a largely conserved resistome under this elevated level highlights the resilience of the poultry GIT microbiome and a high degree of compatibility between dulse-derived bioactive compounds and the resident microbial ecosystem. Future studies should investigate lower, commercially relevant inclusion rates (≤10%) to determine whether comparable microbiome-modulatory effects can be achieved without adversely affecting production performance. In addition, deeper shotgun metagenomic sequencing with strain-level resolution will be required to resolve subtle changes in functional gene repertoires, horizontal gene transfer dynamics, and metabolic pathway activity. Such analysis will be important for robust evaluation of AMR-related gene mobility and for validating the translational potential of dulse as a sustainable functional ingredient in antibiotic-free poultry production systems.

## Conclusion

This study provides the first long-read metagenomic characterisation of the effects of dietary inclusion of dulse (*Palmaria palmata*) in experimental wheat-soyabean meal based diets formulated for AME determination on the caecal bacteriome, resistome, and virulome. Inclusion of dulse at 30%, used as a high experimental sensitivity model, altered microbial composition by enriching beneficial taxa while maintaining overall community stability and a largely conserved resistome profile. The shared resistome, dominated by tetracycline, lincosamide and macrolide resistance genes, is consistent with previously reported persistence of antibiotic resistance genes in poultry gut microbiota, largely unaffected by short-term dietary interventions (Szoke et al., 2025). In contrast, the virulome displayed diet-associated shifts, with dulse inclusion increasing the relative abundance of *Enterobacteriaceae*, primarily *E. coli*, which harbored most of the virulence determinants and their functional repertoire, particularly those involved in adhesion, iron acquisition, and secretion systems. The persistence of both *Col-type* and *Inc-type* plasmids across diets indicates intrinsic potential for horizontal gene transfer within the poultry caecal ecosystem. Overall, our findings demonstrate that high-level dulse inclusion enhances microbial diversity and functional plasticity without concomitant increases in antimicrobial resistance gene abundance. However, the observed modulation of virulence-associated functions highlights the need for careful risk-based evaluation. These findings provide dose-dependent proof-of-principle supporting the role of dulse as a microbiome-modulatory feed ingredient. However, translation to practical application requires validation at commercially relevant inclusion rates (≤10%). Such validation should incorporate comprehensive evaluation of impacts on bird performance, food safety parameters, and antimicrobial resistance dynamics under production conditions.

## Data Availability

The raw long-reads metagenomics sequence (FASTQ files) generated for this study can be found in the NCBI BioProject number PRJNA1406192. The datasets presented in this study can be also found in the article/Supplementary materials. The scripts are stored online on the GitHub project repository: https://github.com/Julio92-C/chickMicro.

## Footnotes

1
https://github.com/Julio92-C/chickMicro/
