# Supplementary material for: Long-reads metagenomics reveals the effects of dulse supplementation on the poultry caecal bacteriome and its associated genetic repertoire
Source: Front Microbiol. 2026 Jul 15;17:1868730. doi: 10.3389/fmicb.2026.1868730 (PMC13416699; doi:10.3389/fmicb.2026.1868730)
Supplement: Supplementary file 4 [file Supplementary_file_1.DOCX]

**Supplementary Data**

**
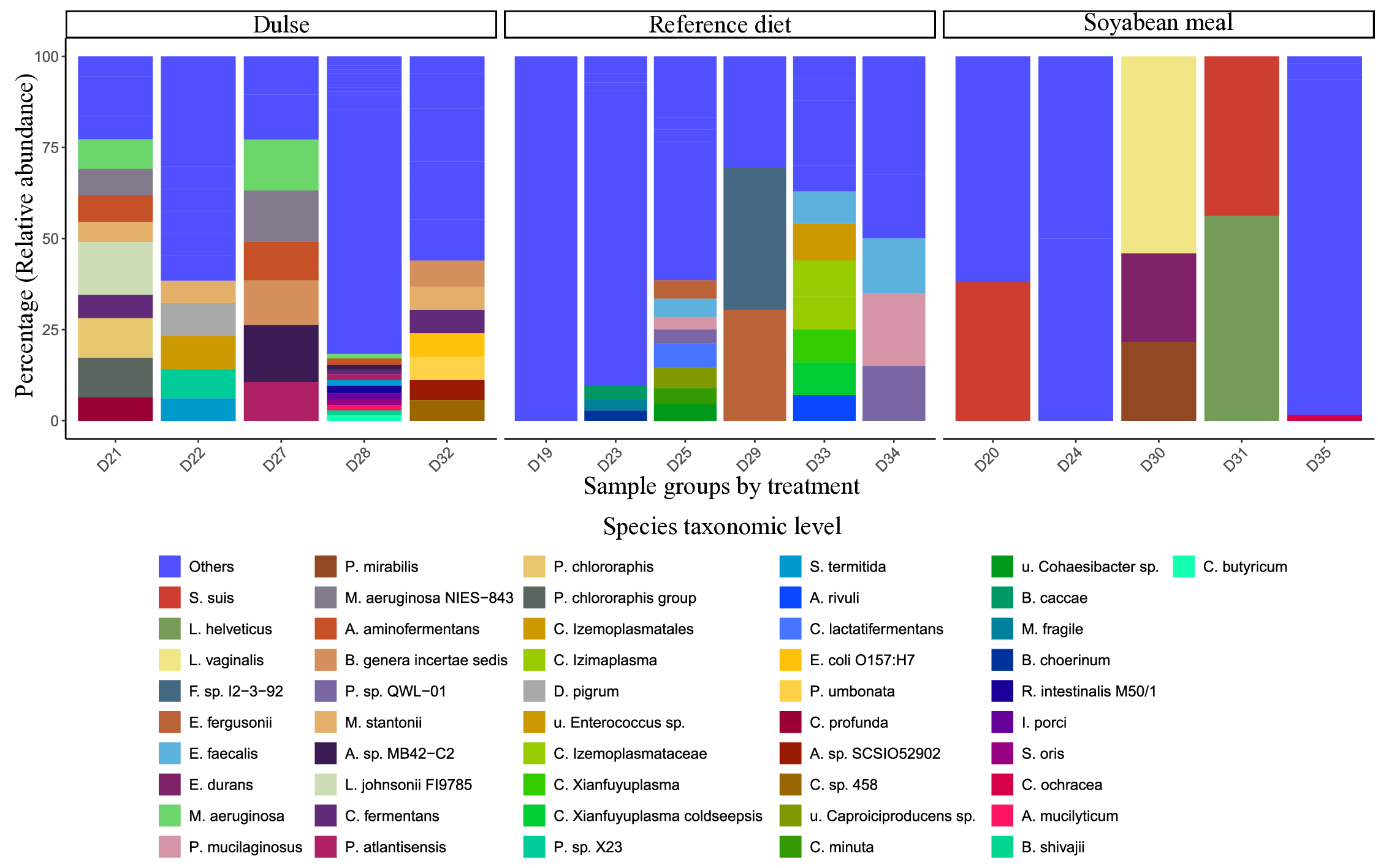
**

**Figure S1.** The figure illustrates the relative abundance of unique taxa across all chicken caecal samples; the y-axis represents the relative abundance at the species level in descending order, and the x-axis represents the sample groups by treatment.

**
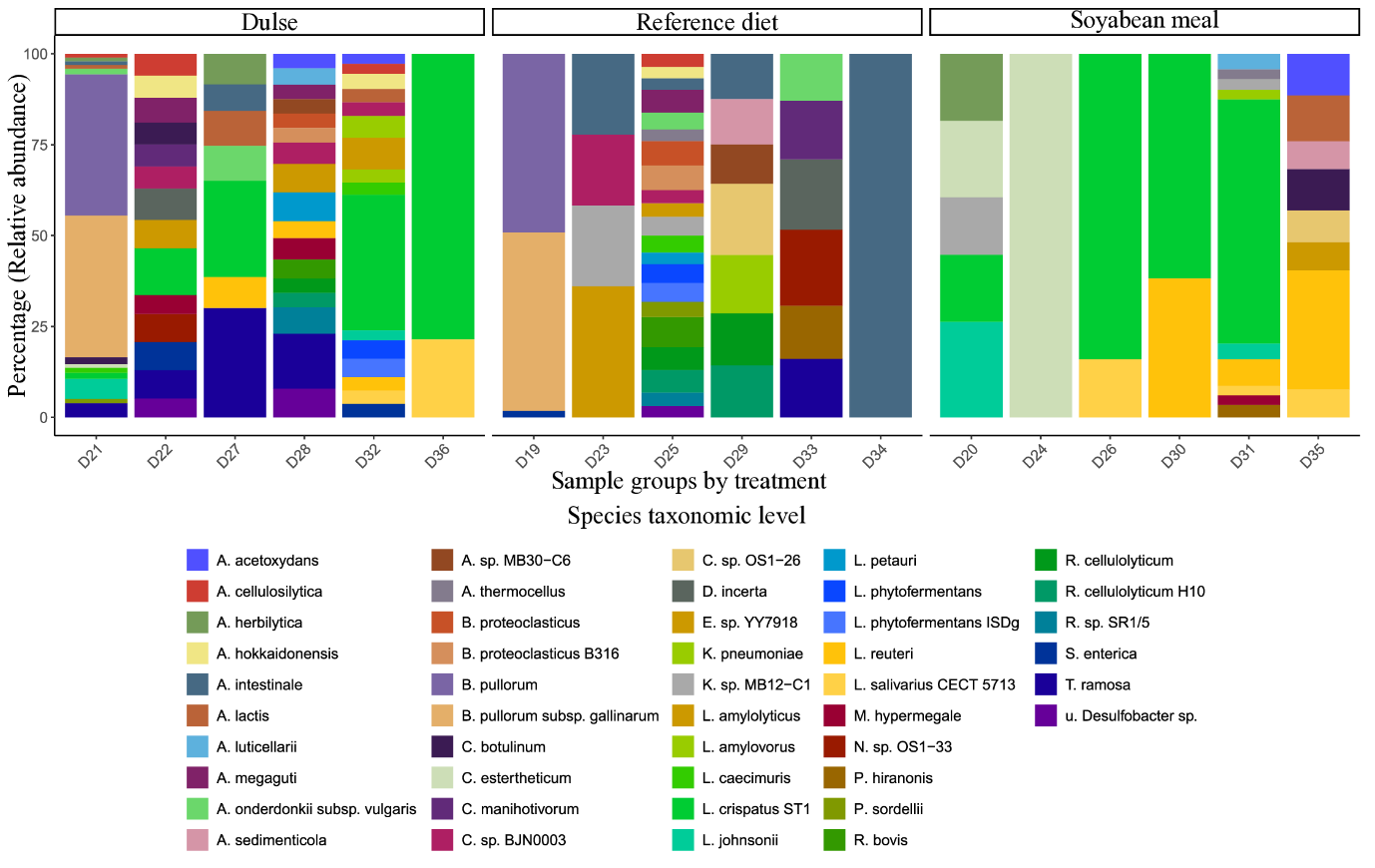
**

**Figure S2.** The figure illustrates the relative abundance of shared taxa across two chicken caecal treatment groups; the y-axis represents the relative abundance at the species level in descending order, and the x-axis represents the sample groups by treatment.

**Table S1.** Summary of the functional groups table at the genus/species level.

| Representative taxa (genus level) | Representative taxa (species level) | Functional role | Primary function |
| --- | --- | --- | --- |
| *Alistipes;*  *Clostridium;*  *Faecalibacterium; Blautia; Bifidobacterium; Ruminococcus; Anaerostipes;*  *Marvinbryantia;*  *Anaerobutyricum;*  *Bifidobacterium* | *A. finegoldii*; *C. scindens*  *A. finegoldii DSM 17242*;  *F. prausnitzii*; *F. plautii*;  *B. obeum*; *B. obeum ATCC 29174*; *B. obeum A2-162*; *B. argi*;  *c*; *B. wexlerae DSM 19850*; *R. lactaris*; *A. hadrus*;  *B. hansenii*; *B. hansenii DSM 20583*; *M. formatexigens*; *M. formatexigens DSM 14469*;  *B. pseudococcoides*;  *F. sp. I3-3-89*; *B. parvula*; *A. hallii*;  *B. producta*; *B. pseudolongum*;  *B. pseudolongum PV8-2*;  *B. animalis*; *B. animalis subsp. animalis* | Primary fermenters / SCFA producers | Carbohydrate and fiber fermentation; butyrate/propionate production; core metabolic backbone. |
| *Lactobacillus / Ligilactobacillus; Bifidobacterium* | *L. salivarius; L. phocaeense;*  *L. crispatus; L. asacharolyticus;*  *B. pseudolongum;*  *B. pseudolongum PV8-2;*  *B. animalis; B. animalis subsp. animalis* | Lactic acid bacteria/probiotics | Carbohydrate fermentation; pH modulation; colonisation resistance |
| *Clostridium;*  *Claveliimonas;*  *Clostridioides; Enterococcus; Escherichia; Enterocloster;*  *Eubacterium;*  *Eubacteriales;*  *Sellimonas* | *C. sp. M62/1*; *C. sp. C1*;  *C. bilis, C. difficile*; *C. asparagiforme*; *E. coli*;  *E. bolteae*; *E. asparagiformis*;  *E. callanderi*; *E. incertae sedis*;  *S. intestinalis, E. faecium, C. perfringens* | Proteolytic/opportunistic taxa and pathobionts | Protein fermentation; potential pathobionts |
| *Ruminococcus; Subdoligranulum; Mediterraneibacter;*  *Clostridium* | *R. torques; M. gnavus; U. Subdoligranulum; S. variabile; C. sp. M62/1* | Mucin/glycan degraders and niche specialists | Mucus/glycan degradation, niche colonisation, cross‑feeding interactions. |
| *Blautia* | *B. hydrogenotrophica; B. hydrogenotrophica DSM 10507* | Hydrogenotrophs / cross‑feeders | H₂ consumption and syntrophic support of fermentation. |

**
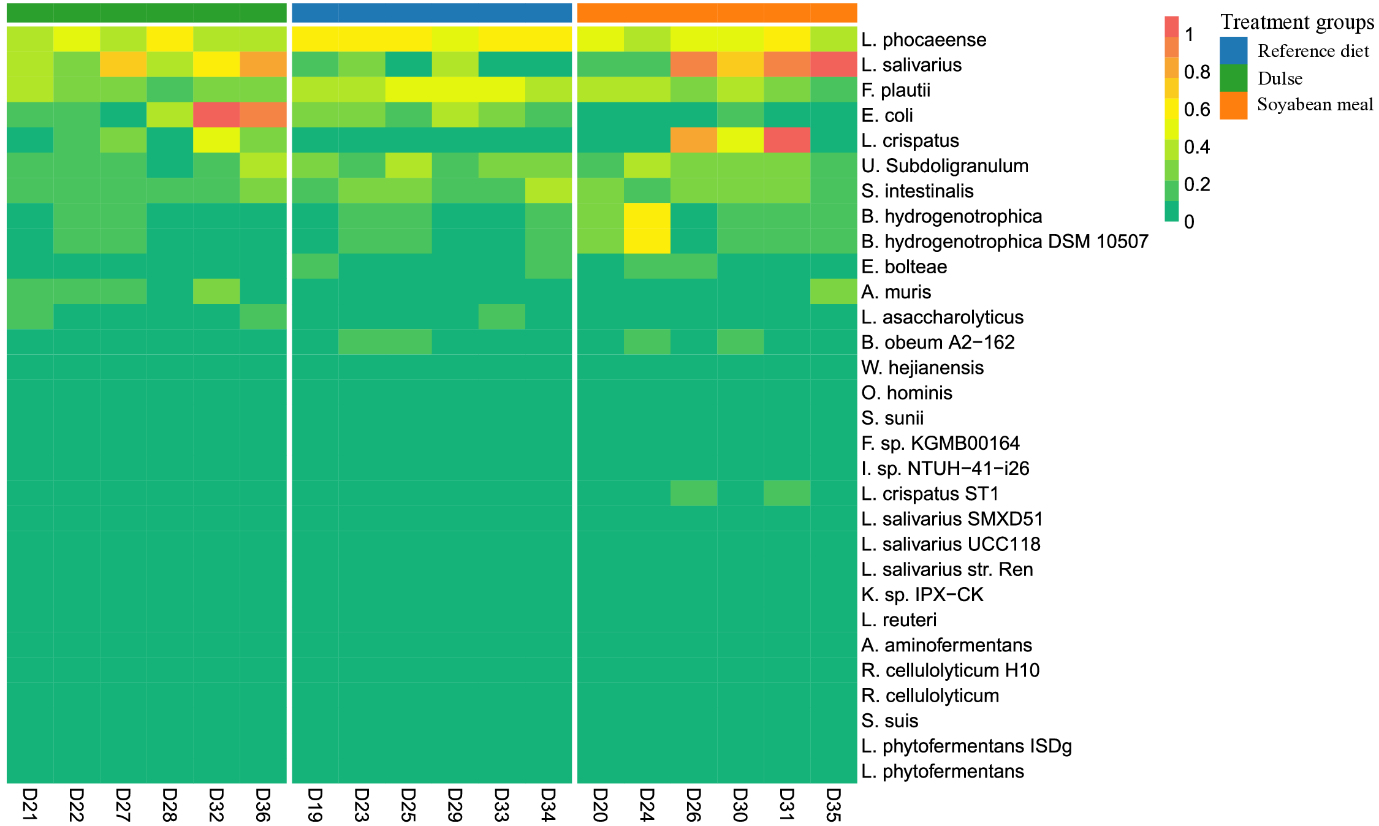
**

**Figure S3.** Relative abundance of the top 30 taxa extracted from the DA analysis.

ARGs profile.

**
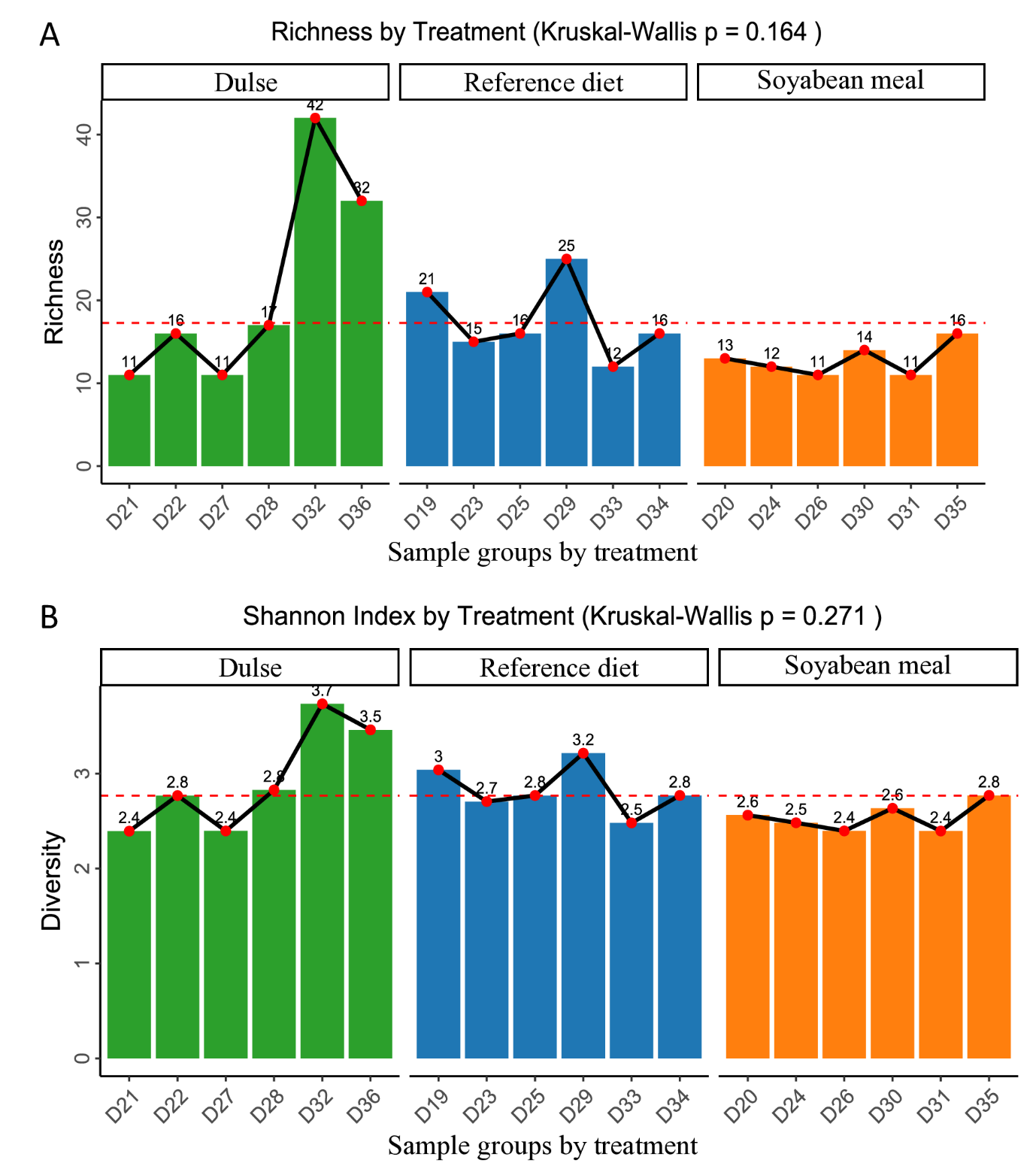
**

**Figure S4.** Alpha diversity metrics measured as Richness **(A)** and **(B)** diversity (Shannon index) of ARG across treatments and over sampling points. Bars and red dots represent the index value per sample. The horizontal red line represents the median. Significance was tested with a Kruskal–Wallis test (p < 0.05). Colours indicated different treatments*.*

**Table S2.** Summary of the host carrying ARGs detected among all treatment groups.

| Related Taxa (Species level) | Antibiotic resistance genes (ARGs) | Antibiotics classes | Pathogenic phenotype |
| --- | --- | --- | --- |
| *C. difficile* | ***APH(3')-IIIa*, *SAT-4, Erm(52)*** | **Aminoglycosides, nucleoside, MLS( macrolides, lincosamides, and streptogramins)** | **Enteric pathogen** |
| *C. perfringens* | ***tet(W)*** | **Tetracyclines** | **Enteric pathogen** |
| *E. coli* | ***TolC*** | **Multidrug** | **Commensal/pathogen** |
| *E. faecium* | ***vanB* gene clusters** | **Vancomycin** | **Nosocomial pathogen** |
| *S. suis* | ***tet(W)*, *lnuC*** | **Tetracycline, lincosamide** | **Zoonotic pathogen** |

**
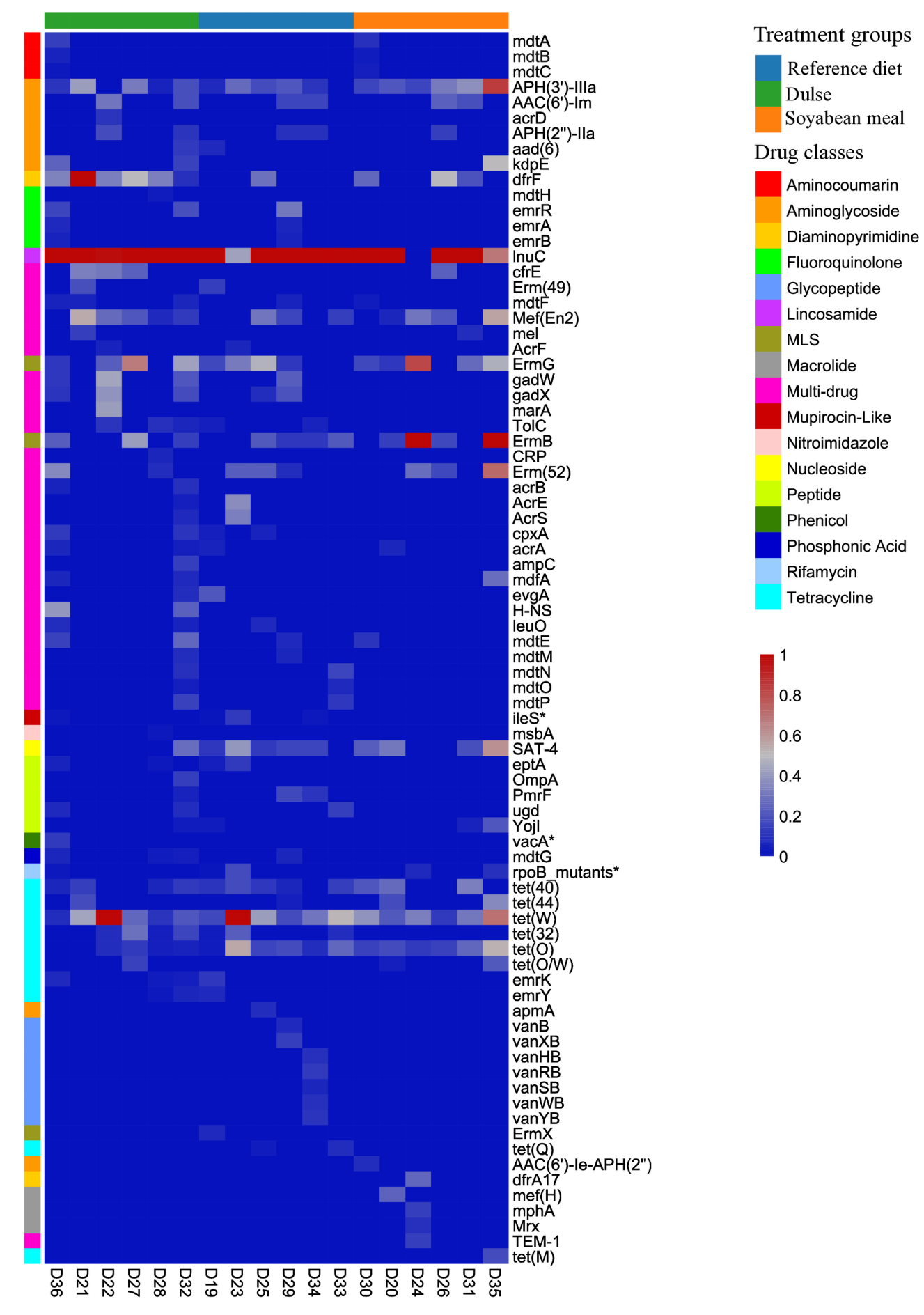
**

**Figure S5.** ARG profile across dietary treatments. This heatmap displays the relative abundance of ARGs across samples collected from chickens fed three different dietary treatments: Reference diet (blue), Dulse (green), and Soyabean meal (orange). Each column represents a sample (D21–D36), and each row corresponds to a distinct ARG. The colour intensity reflects normalised abundance values, ranging from 0 (low abundance) to 1 (high abundance), using a three-colour gradient: blue (Low abundance), white (Intermediate abundance), and red (High abundance).

**
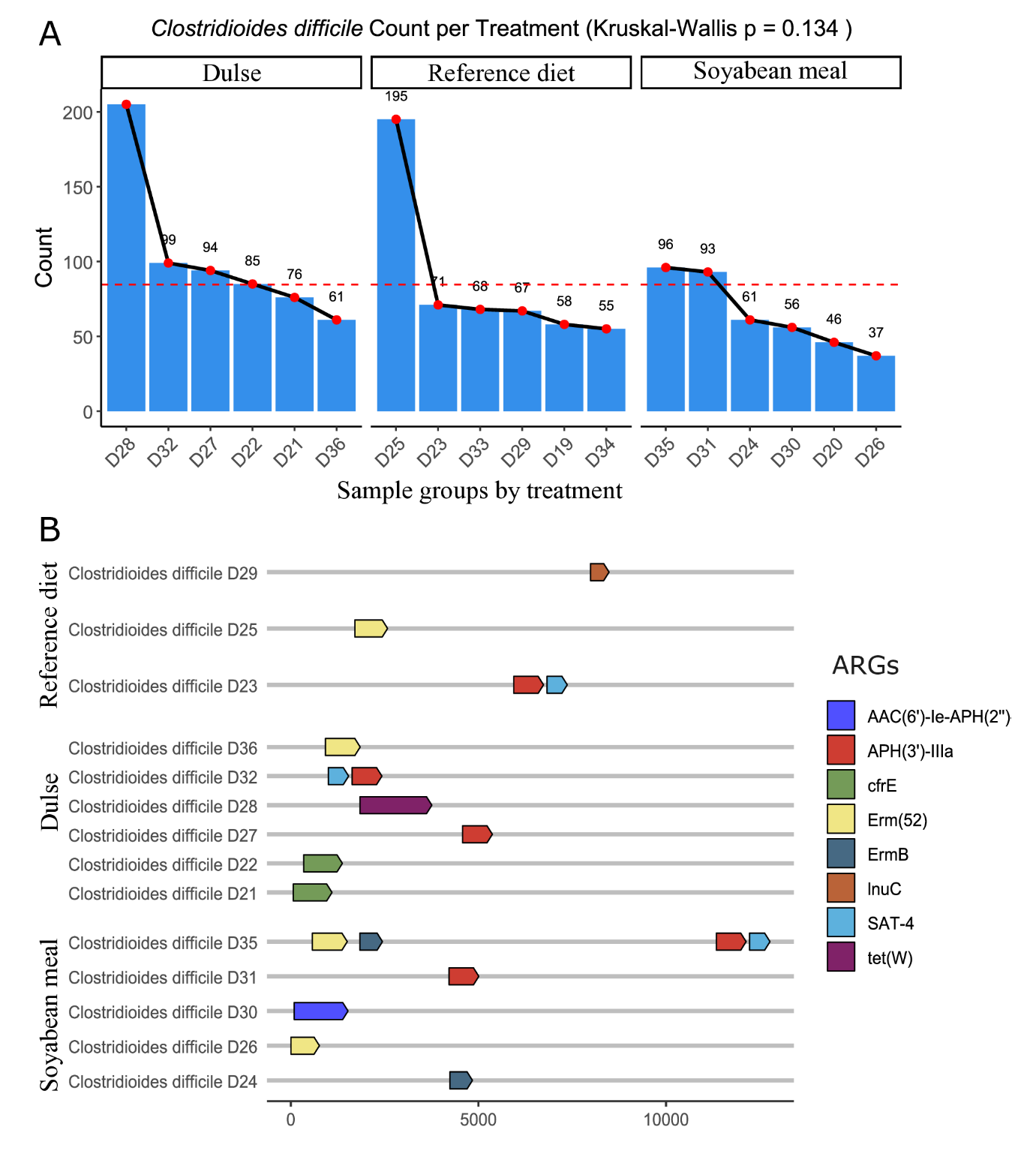
**

**Figure S6.** *Clostridioides difficile*–associated LR-ARG contigs across dietary treatments. **(A)** Bar plot showing the relative abundance of *C. difficile* per sample; the x-axis represents individual samples, and the y-axis indicates observed counts. **(B)** Gene-mapping plot illustrates the diversity and distribution of resistance genes related to *C. difficile* species across the three dietary treatments (Reference, Dulse, and Soyabean Meal).

**
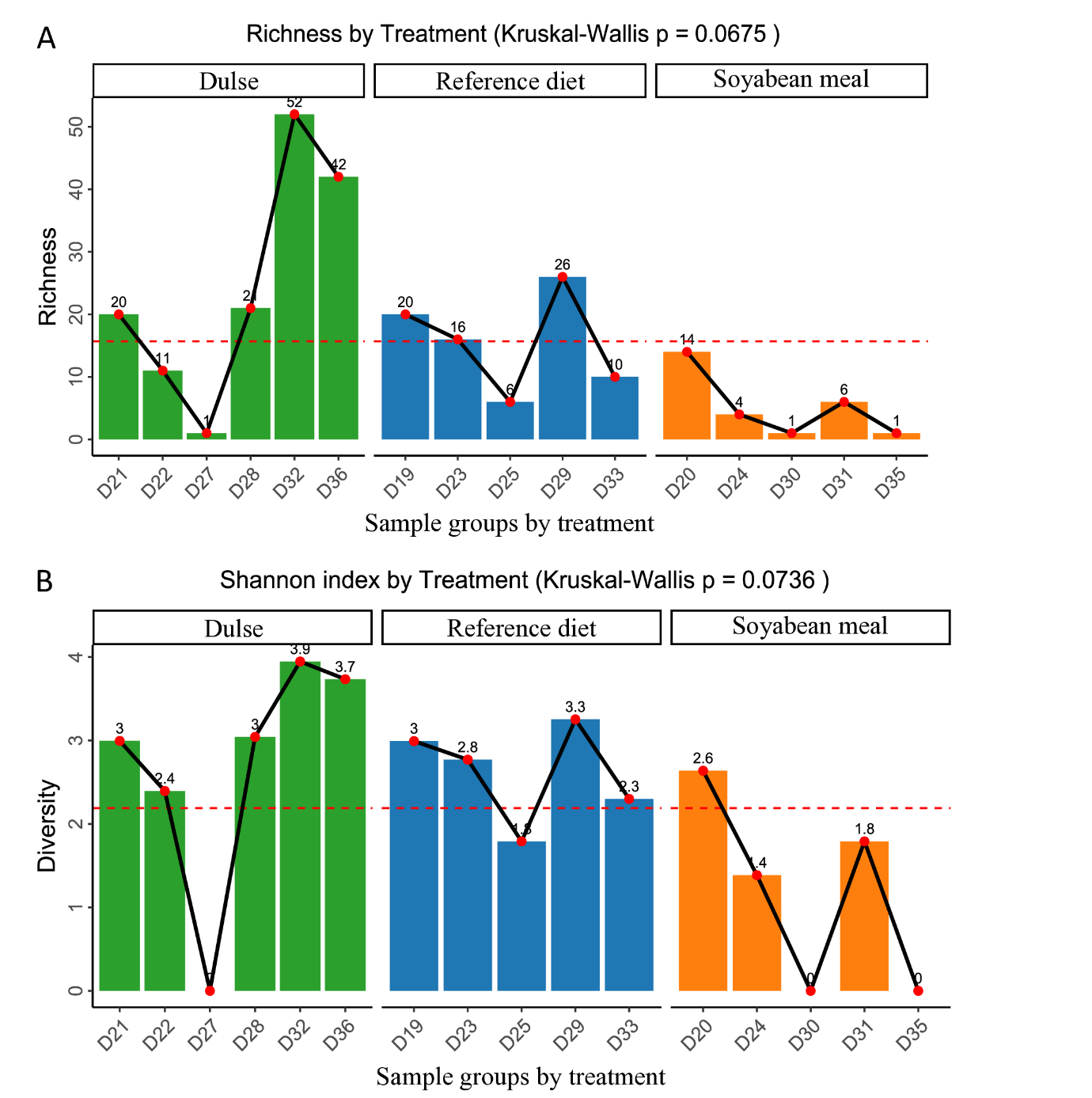
**

**Figure S7.** Alpha diversity metrics measured as Richness **(A)** and **(B)** diversity (Shannon index) of VFs across treatments and over sampling points. Bars and red dots represent the index value per sample. The horizontal red line represents the median. Significance was tested with a Kruskal–Wallis test (p < 0.05). Colours indicated different treatments.

**
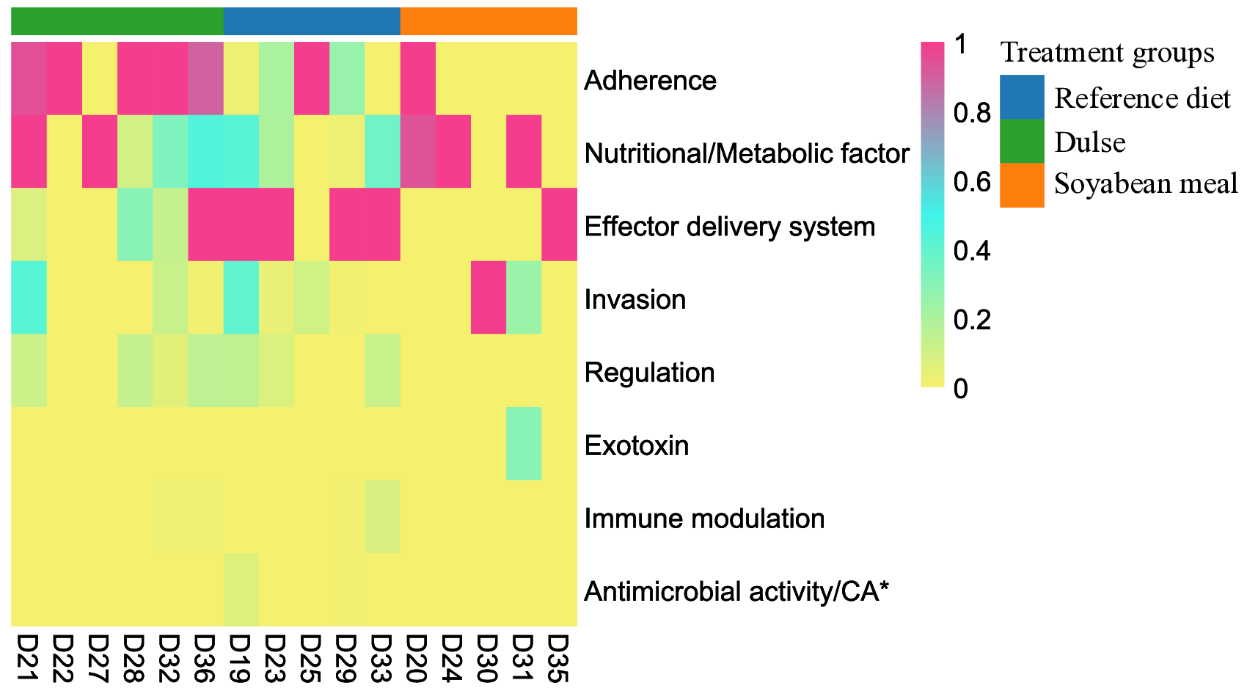
**

**Figure S8.** Heatmap of virulent functions abundance across dietary treatments. Each column represents a sample (D21–D35), and each row corresponds to a distinct virulent function. The colour intensity reflects normalised abundance values, ranging from 0 (low abundance) to 1 (high abundance), using a three-colour gradient: yellow (Low abundance), teal (Intermediate abundance), and Magenta (High abundance).

**
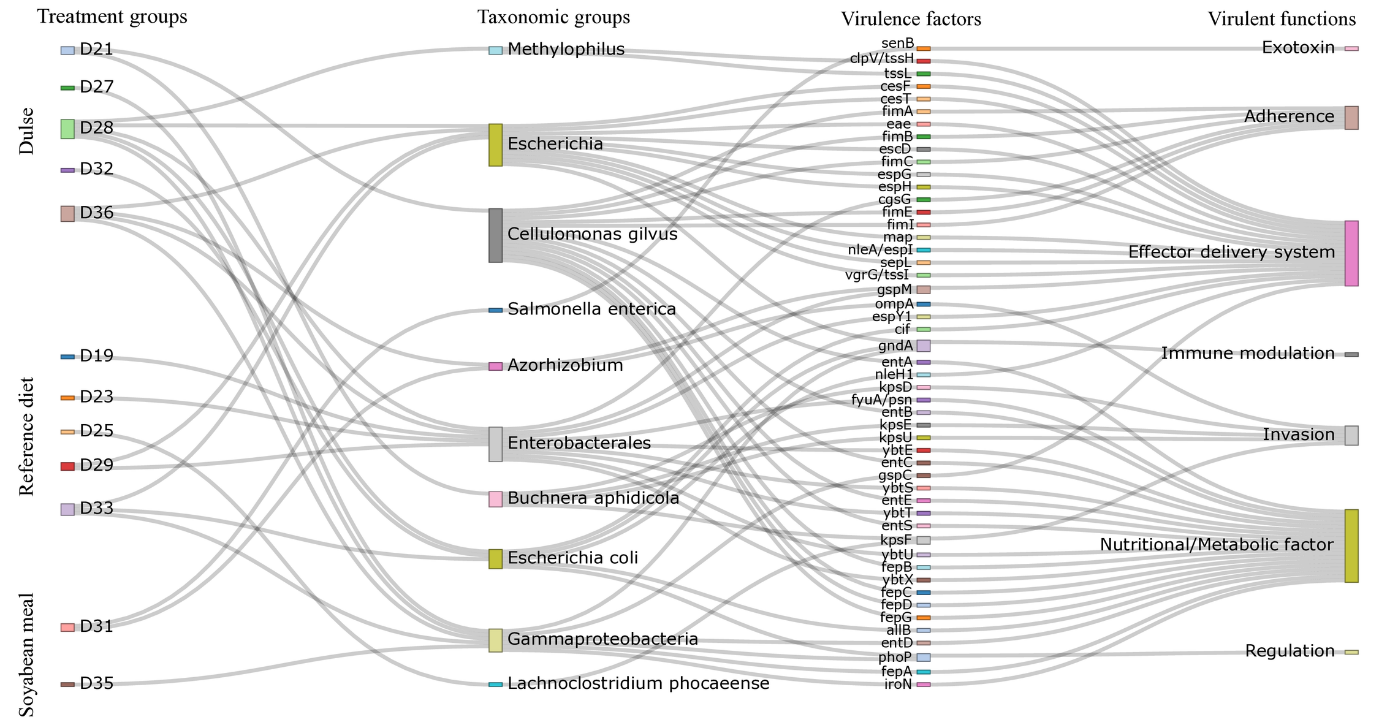
**

**Figure S9.** Distribution and functional mapping of virulence factors within the chicken gut microbiome across dietary treatments. Sankey diagram illustrating the relationships among three distinct treatment groups: Dulse, Reference Diet, and Soyabean Meal. This visualization encompasses a selected array of taxa across multiple taxonomic levels and identifies fifty virulence factors (VFs) along with their associated biochemical functions. The grey strings effectively represent the connections between these components, channelling data from the "Sample Groups" node on the left to the "Functions" node on the right.

**
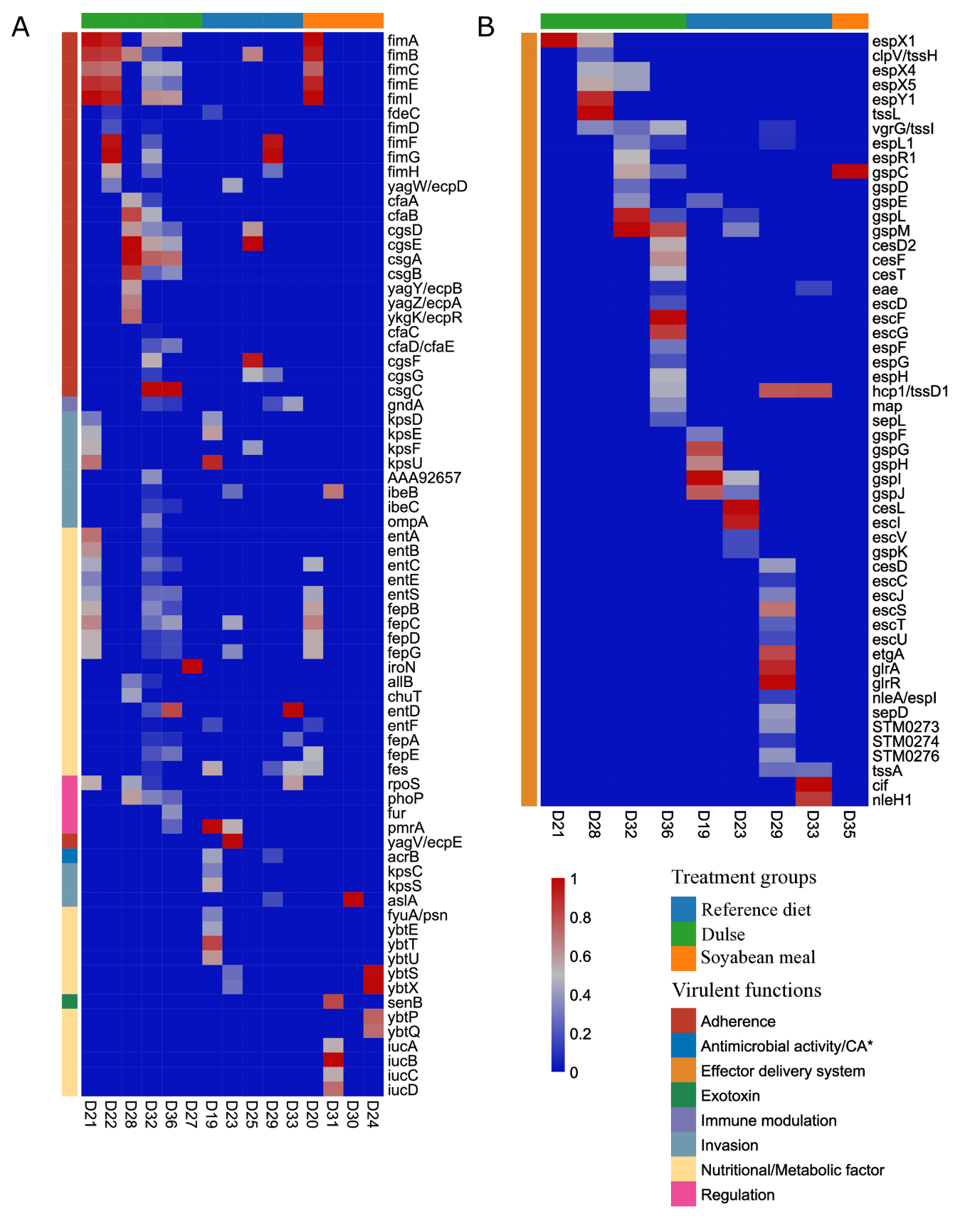
**

**Figure S10.** VFs profile across dietary treatments. Each column represents a sample, and each row corresponds to a distinct virulence gene. The colour intensity reflects normalised abundance values, ranging from 0 (low abundance) to 1 (high abundance), using a three-colour gradient: blue (Low abundance), white (Intermediate abundance), and red (High abundance).

***
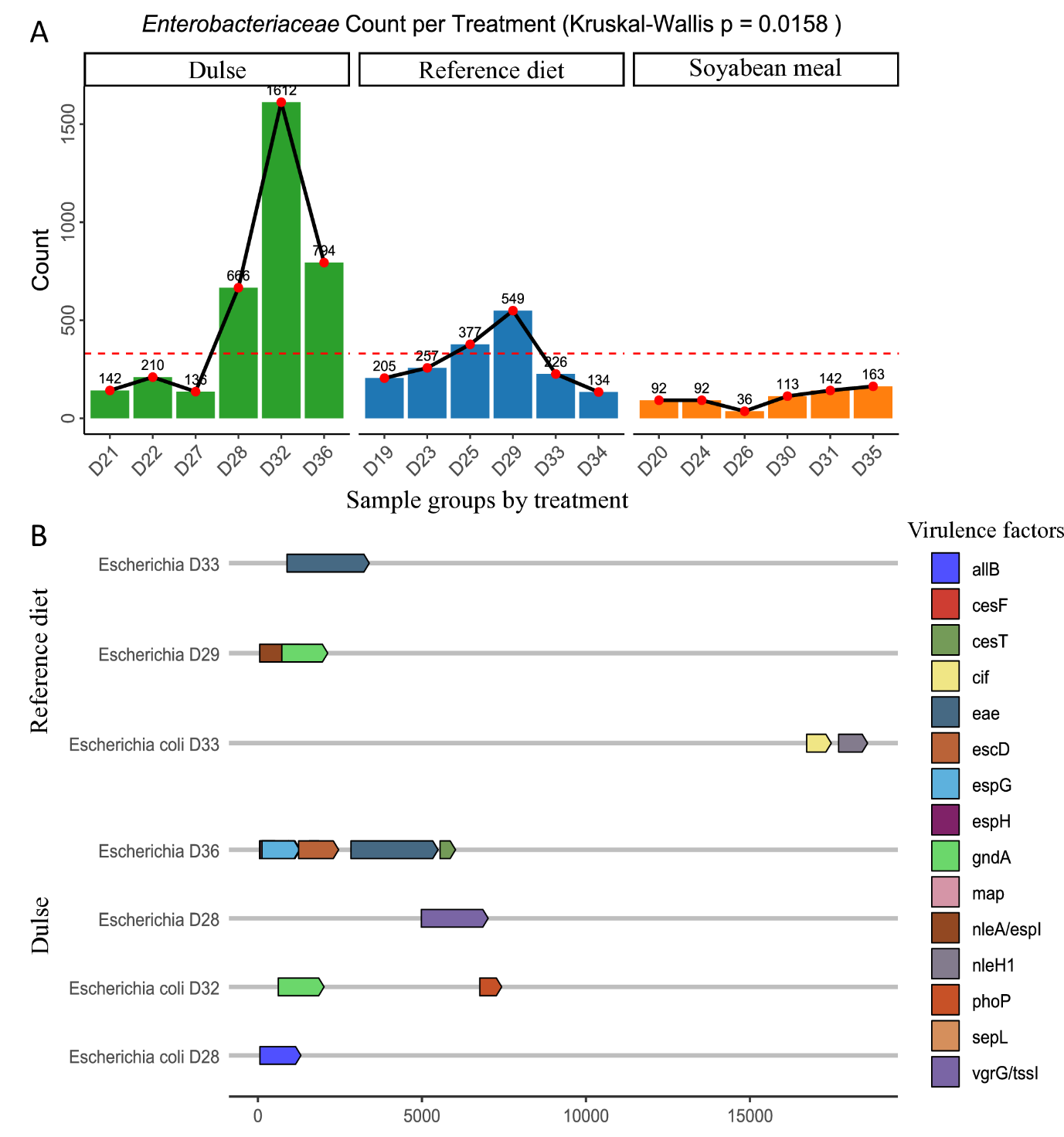
***

**Figure S11. (A)** *Enterobacteriaceae* family–associated LR-VFs contigs across dietary treatments. Bar plot showing the relative abundance of *Enterobacteriaceae* family per sample; the x-axis represents individual samples, and the y-axis indicates observed counts. **(B)** Gene mapping of virulence genes associated with the genus Escherichia (including *E. coli*) within the Reference Diet and Dulse groups, providing further insight into treatment-specific modulation of pathogenic potential.

**
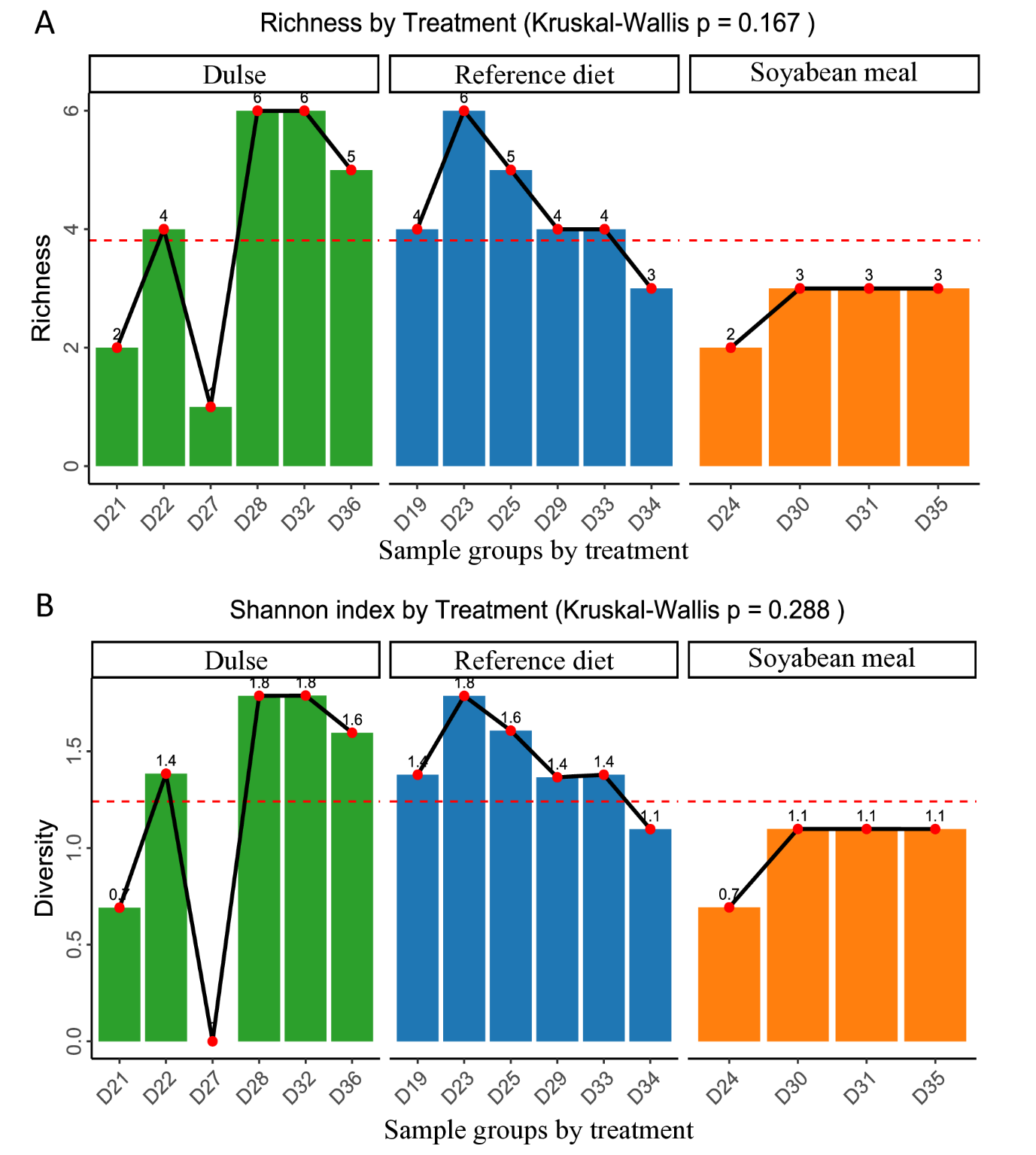
**

**Figure S12.** Alpha diversity metrics measured as Richness **(A)** and **(B)** diversity (Shannon index) of MGEs across treatments and over sampling points. Bars and red dots represent the index value per sample. The horizontal red line represents the median. Significance was tested with a Kruskal–Wallis test (p < 0.05). Colours indicated different treatments.

**
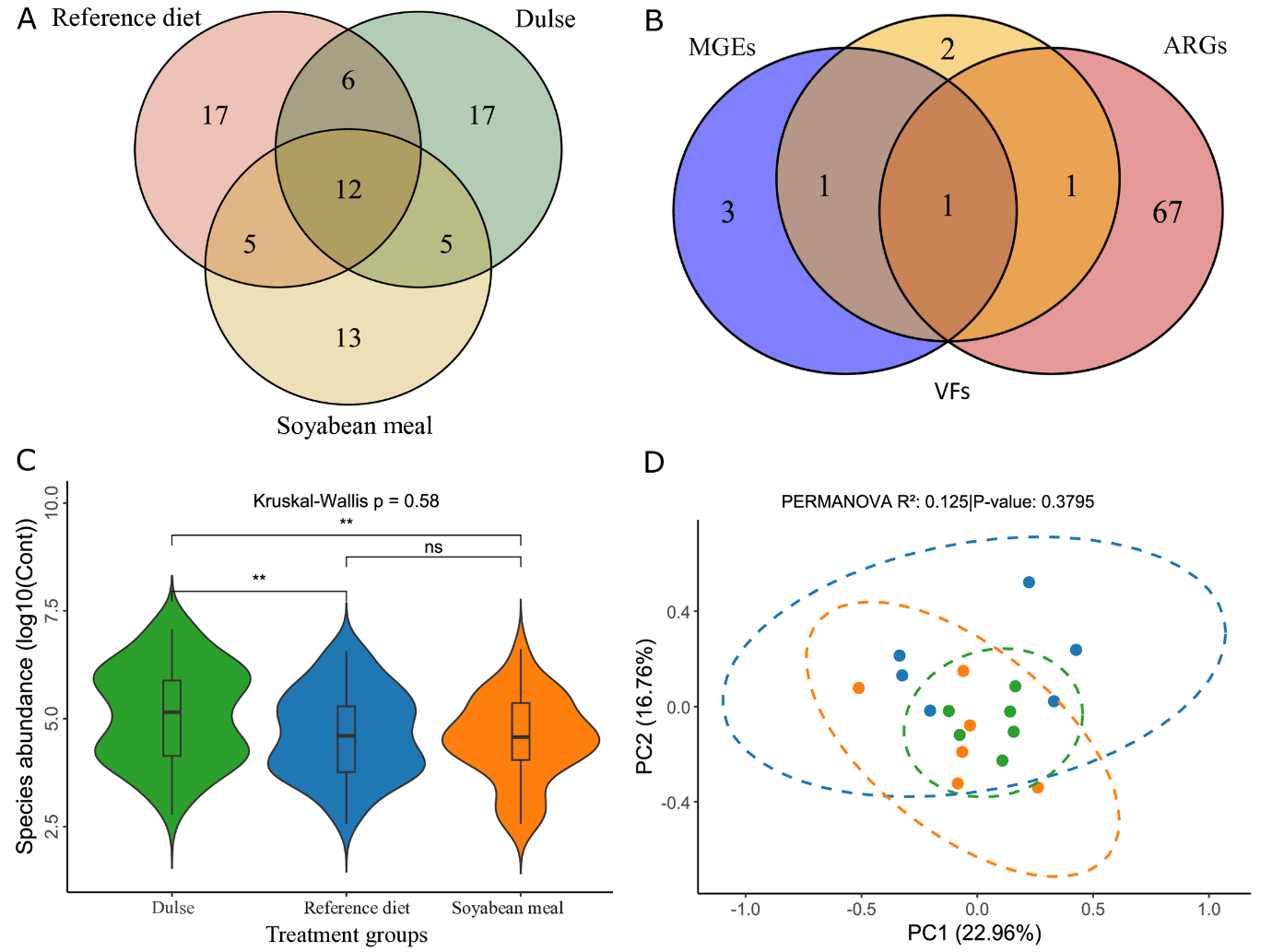
**

**Figure S13**. Microbial community carrying genetic elements at the species levels. **(A)** Overlap of species and GEs **(B)** among treatments, values in each sector correspond to counts of unique/shared features. **(C)** Species abundance (log count) by treatment (Kruskal–Wallis global test). Boxplots are overlaid to show median and interquartile range. Pairwise comparisons are annotated above the plot; asterisks indicate significance levels (Wilcoxon p < 0.05). **(D)** Principal Coordinates Analysis (PCoA) of samples based on Bray–Curtis dissimilarities of specie abundance.
